# A Biosynthetic and Taxonomic Atlas of the Global Lichen Holobiont

**DOI:** 10.1111/1462-2920.70112

**Published:** 2025-06-04

**Authors:** Samantha C. Waterworth, Susan Egbert, John Sorensen, Barry R. O'Keefe, John A. Beutler

**Affiliations:** ^1^ Molecular Targets Program Center for Cancer Research, National Cancer Institute Frederick Maryland USA; ^2^ Department of Chemistry University of Manitoba Winnipeg Manitoba Canada; ^3^ Natural Products Branch, Developmental Therapeutics Program Division of Cancer Treatment and Diagnosis, National Cancer Institute Frederick Maryland USA

**Keywords:** biosynthesis, lichens, metagenomics, natural products, taxonomy

## Abstract

Lichens are pioneer species in several ecosystems, and as such are found in a variety of geographic regions and environments. Here, inspection of metagenomic data from 794 lichen samples from 34 countries reveals the presence of a complex holobiont harbouring remarkable biosynthetic capabilities, particularly in the bacterial consortia, a component that has been somewhat underappreciated. While bacteria were consistently present, their abundance varied among lichen taxa. Common bacterial genera included *Microbacterium*, *Terribacillus*, and *JABEUN01* (an *Acidimicrobiaceae* bacterium awaiting Latin binomial naming assignment) albeit in low abundance. *Lichenihabitans* and *Sphingomonas* genera were moderately abundant, present in approximately 30% of samples, and exhibited an enrichment in the number of biosynthetic gene clusters (BGCs) predicted to encode secondary metabolites (biosynthetic potential). We found that both fungal and bacterial biosynthetic repertoires appeared to follow genus‐specific patterns but that there was greater relative homogeneity of BGCs in the fungal genera. The substantial biosynthetic diversity within lichen holobionts is evident in our findings, with the lichen‐associated bacteria emerging as a promising potential source for sustainable drug discovery.

## Graphical abstract

This analysis of 794 publicly available lichen shotgun metagenomic datasets, covering 467 unique lichen species from 34 countries, revealed that lichens are complex holobionts. They harbour a variety of fungal, algal, and bacterial players with remarkable biosynthetic capabilities. The lichen fungal host taxonomy appears to influence biosynthetic gene cluster (BGC) profiles, particularly in associated bacteria, while fungal partners are more likely to share similar BGCs across multiple genera.

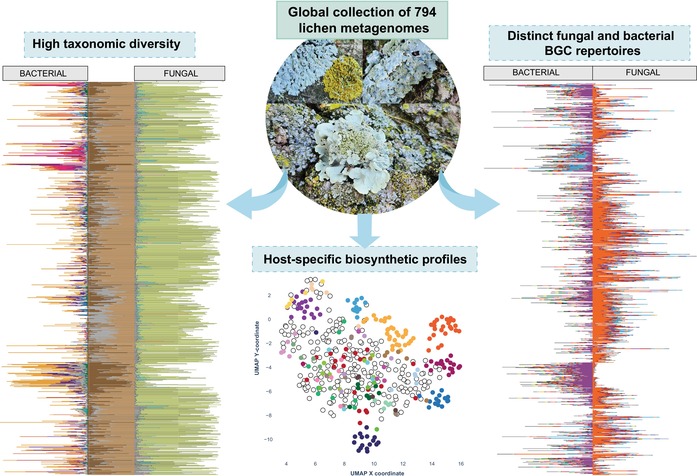

## 1 Introduction

Lichens were traditionally viewed as a bipartite symbiosis between a fungus (mycobiont) and a photosynthetic partner (photobiont), typically an alga (phycobiont or chlorobiont) or cyanobacterium (cyanobiont) (De Carolis et al. 2022). However, more recently it has been recognised that lichens are a multipartite symbiosis of various fungal phyla, algae, and bacterial consortia (Aschenbrenner et al. 2016; Grimm et al. 2021; Morillas et al. 2022; Rolshausen et al. 2023; He et al. 2024) which can be considered “self‐sustaining ecosystems” (Hawksworth and Grube 2020). The bacterial content within a lichen holobiont has been documented between 10^6^ and 10^8^ bacteria per gram of fresh lichen tissue (Cardinale et al. 2008; Grube et al. 2009; Bjelland et al. 2011). While relatively little is known about the role of non‐cyanobacteria associated with lichen holobionts (Grimm et al. 2021; He and Naganuma 2022), some bacteria have been reportedly involved in various roles to support the holobiont, such as nutrient cycling, stress mitigation, nutrient scavenging, degradation of phenolic compounds, and production of vital molecules such as vitamins and hormones (Grube et al. 2009, 2015; Sigurbjörnsdóttir et al. 2014; Cernava et al. 2017).

While over 1000 known bioactive compounds have been isolated from lichen holobionts, only seven have been linked to putative biosynthetic gene clusters (BGCs), and of those only four (atranorin, lecanoric acid, grayanic acid, and gyrophoric acid) have been experimentally validated as originating from the fungal host (Armaleo et al. 2011; Kealey et al. 2021; Kim et al. 2021; Singh et al. 2022a; Singh 2023). Further, a handful of pharmaceutically interesting compounds have been isolated from culturable lichen‐associated bacteria (primarily *Actinomycetes*) including novel angucyclines (Motohashi et al. 2010), coumabiocins (Cheenpracha et al. 2010), and uncialamycin (Davies et al. 2005). However, limitations in culturing fastidious lichen‐associated bacteria can greatly affect the outcome of such searches, limiting our understanding of the biosynthetic potential of these consortia (Biosca et al. 2016; Zakeri et al. 2022; Hou et al. 2023; Weeraphan et al. 2023). An early survey of PKS genes present in lichens by Davies and colleagues indicated that there is a large biosynthetic diversity potentially residing in the *Cyanobacteria* in the lichen holobiont (Miao et al. 2001). With such evidence for bioactive promise, along with facile genomic data generation and analysis, and the option of heterologous expression in other more tractable bacterial hosts, the potential for discovering novel compounds seems vast. Indeed, several BGCs have been bioinformatically predicted in several individual lichen mycobionts (Bertrand and Sorensen 2018; Bertrand et al. 2018a, 2018b; Calchera et al. 2019; Shishido et al. 2021; Singh et al. 2021; Singh et al. 2022b), and it would be advantageous to know which lichens hold the greatest potential for chemical novelty and whether some lichen‐derived compounds may be of bacterial origin.

In this study, we mapped the biosynthetic potential of bacterial and fungal organisms in 794 lichen metagenomes and recovered 22,713 fungal BGCs and 30,059 bacterial BGCs, of which 4265 (18.7%) and 3486 (11.6%) were predicted to be complete, respectively. Most bacterial BGCs were predicted to occur in the *Pseudomonadota*, *Acidobacteria*, and *Actinomycetes* phyla, taxa that also appeared to be most abundant in the lichen samples. The vast majority of fungal BGCs appeared to originate from the dominant mycobiont, but secondary fungal partners also contribute to the overall biosynthetic capacity of the holobionts. Similarity networks of all detected BGCs suggest an enormous biosynthetic potential in both bacteria and fungi within lichens. While the biosynthetic potential of lichenized fungi has been acknowledged (Singh et al. 2025), the biosynthetic wealth of the entire holobionts, in particular the bacterial component, is currently underappreciated and offers an avenue for natural product bioprospecting and drug discovery. As this report investigated the biosynthetic potential of only a small portion of the estimated > 20,000 lichen species, it both represents the most comprehensive analysis to date and highlights a significant opportunity for further discovery.

## 2 Results and Discussion

### 2.1 Overview of Datasets Included in the Study

A total of 794 publicly available lichen metagenome datasets were successfully assembled and passed quality control criteria (please see Section 3 for full criteria). All lichen holobionts were taxonomically classified within the phylum *Ascomycota* and included 10 classes, 30 orders, 70 families, 182 genera, and 467 unique species, collected from 34 countries (Figure S1, Dataset 1). The order *Lecanorales* appears over‐represented in available online datasets, comprising 466 of the samples (58.7%). At a genus level, there is an over‐representation of metagenomes in the online databases representing the genera *Cladonia*, *Rhizoplaca*, *Xanthoparmelia*, and *Lecanora*, accounting for 114 (~14%), 73 (~9%), 61 (~8%), and 58 (~7%) of the samples, respectively. This over‐representation in the available data resulted from several population genomics studies (Bradshaw et al. 2020; Ivanovich et al. 2022; Keuler et al. 2022; Zhang et al. 2022; Hoffman et al. 2023; Leavitt et al. 2023). Similarly, there was a significant overrepresentation of metagenomic sequence datasets of lichen collected in the United States, accounting for 611 (~77%) of all samples, with samples collected from Canada, Spain, and Japan accounting for 49 (~6%), 23 (~3%), and 16 (~2%) of all samples, respectively. This highlights an opportunity for wider collection and metagenomic sequencing of lichen samples from the continents of Africa, South America, Asia, Australia, and other countries within the Oceania and Polar regions.

### 2.2 Taxonomic Distribution Within Lichen Holobionts

**FIGURE 1:**
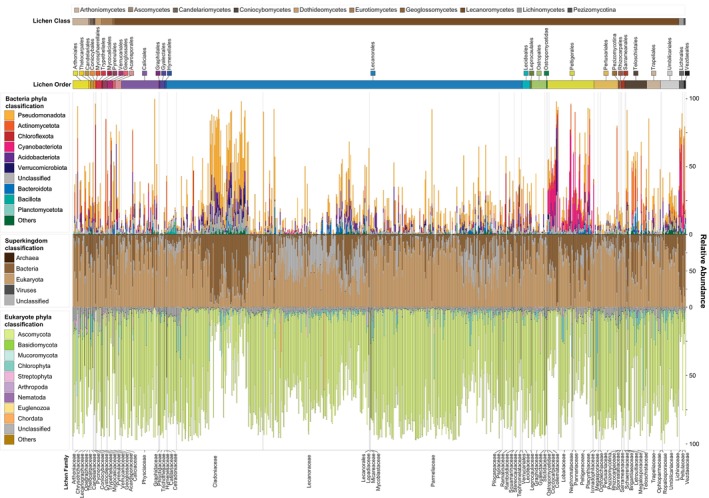
The relative abundance of lichen‐associated taxa in all samples at a kingdom level (centre), and a phylum level for all eukaryotic (bottom) and bacterial contigs (top) that were greater than 3000 bp in length. Each stacked bar represents a lichen holobiont sample, and all samples are organised by hierarchical classification of the lichen sample from Class through to species. Classification to the level of Family is indicated. Coloured keys are provided on the top for lichen sample Class and Order. The top 10 most abundant bacterial and eukaryotic phyla are indicated with a coloured key on the left. All other phyla were summed into “Others” in each case. Note that the phyla bars for the bacterial and eukaryotic kingdoms are effectively the kingdom bars flipped outward and subsequently divided and recoloured by phyla.

Evaluation of contig cumulative length, count, and relative abundance before and after excluding contigs smaller than 3000 bp revealed that most small contigs were “Unclassified” (Figure S2, Dataset 2). This is not unexpected as the smaller a contig, the less taxonomically useful information is available for a successful taxonomic assignment. We then considered only contigs greater than 3000 bp (Dataset 3). We found that contigs classified within the *Eukaryote* superkingdom were most abundant (average relative abundance of 60.3% across all samples), with bacterial and unclassified contigs present at average relative abundances of 25.2% and 14.4%, respectively, across all samples (Figure 1). Contigs classified as viral or archaeal were present at an average abundance across all samples of 0.002% and 0.005%, when considering all contigs (Dataset 2), and 0.02% and 0.03%, respectively, when considering only contigs greater than 3000 bp in length (Dataset 3).

We detected at least 47 different eukaryotic phyla across all lichen samples. All lichens in this study were classified within the phylum *Ascomycota*, so it was not unexpected that most eukaryotic contigs in all lichen metagenomes were classified within the *Ascomycota*. Phyla *Chlorophyta* and *Streptophyta* were detected in 727 (92%) and 721 (91%) of all lichen samples, respectively (Figure 1, Table 1). *Chlorophyta* was the more abundant of the two, with a mean abundance of 2.9% relative to a mean *Streptophyta* abundance of 0.5% across all samples. This is in keeping with historical observations wherein nearly all lichen algae (phycobionts) are classified within the *Chlorophyta* (Sanders and Masumoto 2021). These algal contigs were presumed to represent the dominant photobionts in each sample. Similarly aligned with more recent discoveries that more than one fungus may be present in a given lichen holobiont (Grimm et al. 2021; Sanders and Masumoto 2021; Tagirdzhanova et al. 2021), we noted that there were 533 samples (67.1%) with *Basidiomycota* and 276 samples (34.7%) with contigs classified as *Mucoromycota* (Table 1).

**TABLE 1:** The average relative abundance and presence of all contigs > 3000 bp were classified within the top 10 phyla of bacterial and eukaryotic contigs in all lichen samples.

	Present in N samples	Average abundance across samples
Bacteria		
Pseudomonadota	765	9.36
Acidobacteriota	610	2.66
Actinomycetota	623	1.86
Cyanobacteriota	471	1.81
Bacteroidota	519	0.61
Chloroflexota	389	0.42
Verrucomicrobiota	367	0.42
Planctomycetota	366	0.16
Bacillota	557	0.14
Others	579	0.47
Unclassified	777	3.2
Eukaryota		
Ascomycota	793	61.66
Chlorophyta	727	2.94
Streptophyta	721	0.45
Chordata	262	0.15
Basidiomycota	533	0.09
Arthropoda	340	0.07
Mucoromycota	276	0.01
Nematoda	88	0.01
Euglenozoa	32	0
Others	402	0.03
Unclassified	794	3.15

We found 101 different bacterial phyla across all lichen samples. Lichens classified within orders *Peltigerales* and *Lichinales*, and families *Letrouitiaceae*, *Cladoniaceae*, and *Verrucariaceae*, were particularly rich in bacteria (Figure 1, Dataset 4). The most abundant and ubiquitous bacterial phyla were *Pseudomonadota* (syn. *Proteobacteria*), *Acidobacteriota*, *Actinomycetota*, and *Cyanobacteriota* (Figure 1, Table 1). The average abundances of the bacterial phyla highlight the significant presence of bacteria in lichen holobionts, showing they are not merely minor members of the holobiont community. Cyanobacteria were particularly abundant in lichens classified within the *Peltigerales* and *Lichinales* orders (Figure 1), likely representing the cyanobionts characteristic of these orders (Graham et al. 2018; Jung et al. 2021). While it is conceivable that some bacteria present in these datasets may represent contamination from transient or environmental bacteria (e.g., soil‐borne bacteria), the findings here correlate with trends observed across several studies, wherein *Pseudomonadota* is found as the most dominant bacterium, with *Actinomycetota* generally found to be the second most dominant in lichen holobionts (Bates et al. 2011; Mushegian et al. 2011; Sierra et al. 2020; Noh et al. 2021; He and Naganuma 2022; Weeraphan et al. 2023). Further, the fact that these trends have persisted across ~800 samples implies that the effect of contaminating environment‐borne bacteria would have a minimal impact, if any, on the overall result.

### 2.3 Binning of Bacterial Contigs Into Putative Metagenome‐Assembled Genomes

Bacterial contigs were clustered into metagenome‐assembled genomes (MAGs). A total of 2842 MAGs were recovered from 448 (56.4%) of the 794 lichen metagenomes, of which 339 (11.9%) were of high quality and 1091 (38.4%) were of medium quality (Figure S3A, Dataset 5) per MiMAG reporting standards (Bowers et al. 2017). Failure to recover MAGs from ~43% of metagenomes may be due to lower sequencing depth, low bacterial abundance, high bacterial diversity, strain heterogeneity, a lack of marker genes for anchoring binning, or any combination of these factors per lichen metagenome (Bharti and Grimm 2019; Miller et al. 2019; Liu et al. 2022; Meyer et al. 2022; Orellana et al. 2023; Rees et al. 2023). Considering only the high and medium‐quality bins (*N* = 1430), the majority of bins were classified within phyla *Pseudomonadota* (*N* = 681) and *Acidobacteriota* (*N* = 359) (Figure S3B). At the level of genus, *EB88* (*Acidobacteriaceae*) (*N* = 101) and *LMUY01* (*Acetobacteraceae*) (*N* = 70) genera were most commonly recovered and were found in 90 (11.3%) and 52 (6.5%) of the lichen samples, respectively.

**FIGURE 2:**
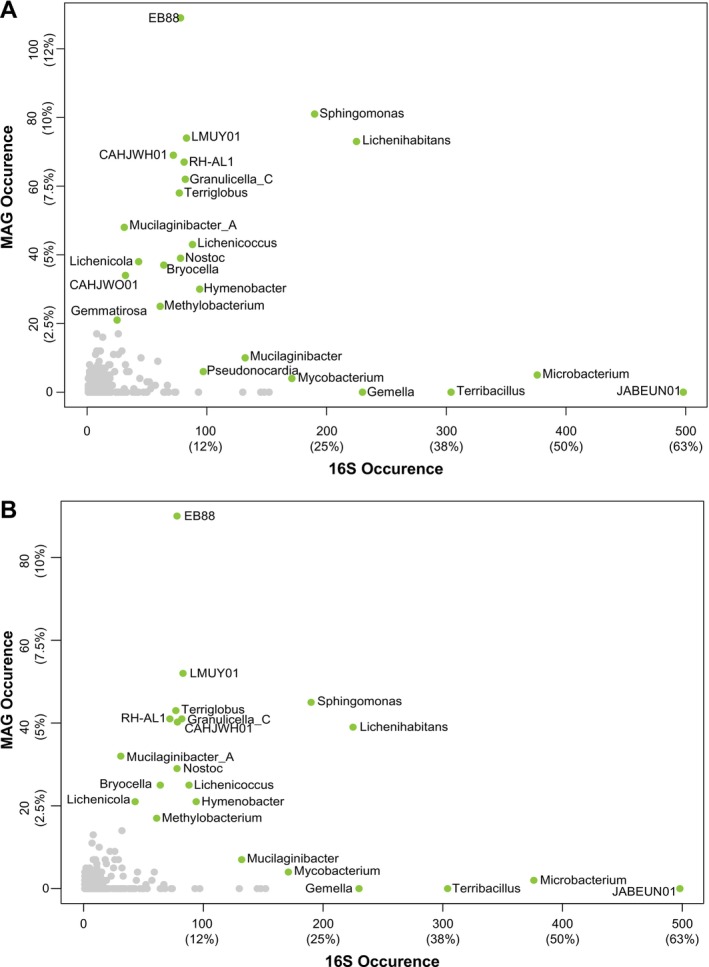
Comparison of genera occurrence counts in MAG and 16S rRNA data sets from the 794 lichen metagenomes. (A) Comparison using all MAGs. (B) Comparison with only high and medium quality MAGs. Genera with high occurrence counts have been labelled and coloured green. Occurrence has been presented as both counts and as a percentage (out of 794 samples) on both the x and y axes.

A study by Tagirdzhanova et al. (2024) describes the genus *Lichenihabitans* as the most commonly recovered and detected in 67.5% of the metagenomes they surveyed. In the present study, 46 MAGs were classified as *Lichenihabitans* and were present in 4.9% of metagenomes. To be fully comparable to the study conducted by Tagirdzhanova et al. (2024), we extracted all 16S rRNA genes (*N* = 19,044) from the metagenomes and taxonomically classified them using IDTAXA (Murali et al. 2018), as performed by Tagirdzhanova and colleagues (Dataset 6). Recovered 16S rRNA gene sequences ranged from 400 bp to full length (~1500 bp) (Figure S4A), and the choice of sequencing technology did not appear to influence the number of 16S rRNA gene sequences recovered (Figure S4B). However, IDTAXA only successfully classified 6440 (33.8%) of the 16S rRNA sequences at the level of *Bacteria*. To overcome this limitation, we aligned the 16S rRNA gene sequences against the GTDB 16S rRNA gene database using blastn (Camacho et al. 2009). Approximately 49.1% of the 16S sequences could be classified to the genus level (%ID > 95%; Yarza et al. 2014) (Dataset 6). The most commonly recovered genera were *JABEUN01* (*Acidimicrobiaceae*), *Microbacterium* (*Microbacteriaceae*), and *Terribacillus* (*Amphibacillaceae*), occurring in 498 (62.7%), 376 (47.4%), and 304 (38.3%) of the 794 lichen metagenomes, respectively (Dataset 6). Please note that the *JABEUN01* genus does not follow the traditional Latin binomial naming format. This is because the GTDB database, on which classifications are based, uses a strain identifier as a placeholder when there is no existing genus name or a binomially named representative genome. The *Lichenihabitans* genus, postulated to be a ubiquitous member of lichen holobionts (Tagirdzhanova et al. 2023, 2024), was detected in 225 (28.3%) of the lichen metagenomes. Quality MAG recovery often reflects bacterial abundance in a given metagenomic sample (Sangwan et al. 2016; Papudeshi et al. 2017; Royalty and Steen 2019). Therefore, using MAG abundance as a proxy for genus abundance and 16S rRNA gene occurrence as a proxy for ubiquity, we found that *Lichenihabitans* and *Sphingomonas* genera appear both abundant and ubiquitous among lichen holobionts whether all MAGs are considered (Figure 2A) or if only high and medium quality bins are considered (Figure 2B).

### 2.4 Biosynthetic Potential in Lichen Holobionts

A total of 52,772 BGCs were recovered; 30,059 bacterial BGCs from 589 (74.2%) of the lichen samples, and 22,713 fungal BGCs from 777 (97.9%) of the lichen samples (Dataset 7). Of these, 3486 (11.6%) bacterial BGCs and 4265 (18.8%) fungal BGCs were predicted to be complete (Figure S5A). Please note, “completeness” of a BGC is predicted by the antiSMASH algorithm based on whether expected core genes of a given BGC type can be found on the same contig within the predicted BGC boundary. The number of BGCs identified in each dataset will be influenced by factors such as sequencing depth, holobiont diversity, assembly fragmentation, genome size, and the presence of known biosynthetic motifs. Therefore, these counts reflect general trends across lichen holobionts rather than definitive totals. Furthermore, the significant difference in genome size between bacterial and fungal partners will likely impact BGC recovery. Larger fungal genomes are more prone to incomplete assembly, potentially leading to an underestimation of their biosynthetic capacity. Conversely, the greater biomass of the fungal partner in samples may result in insufficient sequencing coverage to fully capture the biosynthetic potential of less abundant bacterial taxa. When corrected for sequencing depth (Dataset 8), there were an average of 0.05 bacterial and 0.1 fungal BGCs per megabase of total sequence (Figure S5B), which increased to 0.2 bacterial and 0.3 fungal BGCs when adjusting for the sequencing depth of contigs > 3000 bp (Figure S5C). Among lichen genera, 19 had at least 1 bacterial BGC, and 23 had at least one fungal BGC per megabase of sequence (Figure S5D), highlighting them as potential targets for drug discovery. Additionally, it was intriguing to note that lichen genera tended to either be rich in bacterial or fungal BGCs (per megabase of sequence) but not both (Figure S5D). This may indicate that lichen holobionts may rely more on different players in the holobiont for secondary metabolite production.

**FIGURE 3:**
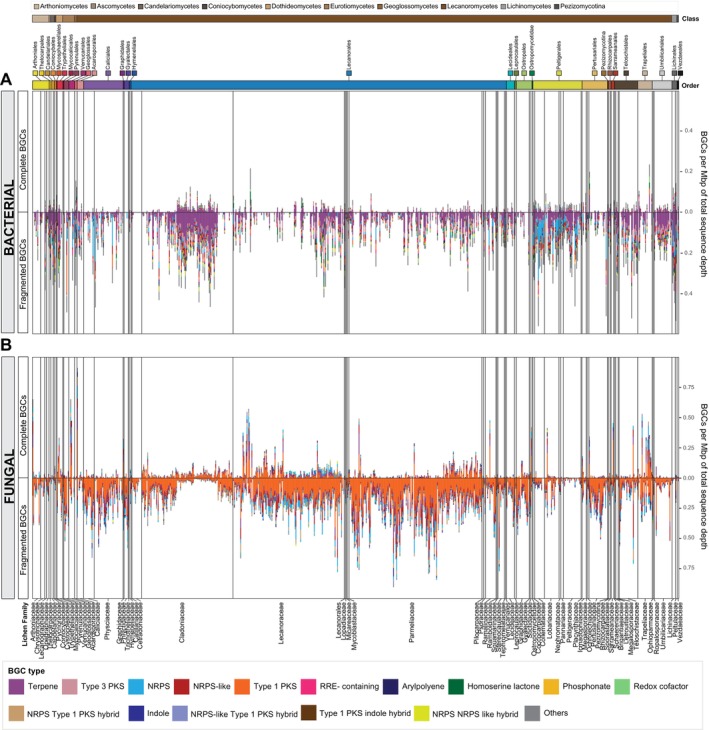
The distribution of bacterial and fungal BGCs detected in all lichen holobiont samples. (A) The summed number of complete and fragmented BGCs per Mbp of total sequence depth per sample categorised by BGC type from bacterial contigs. (B) The summed number of complete and fragmented BGCs per Mbp of total sequence depth per sample categorised by BGC type from fungal contigs. Each stacked bar represents a lichen holobiont sample, and all samples are organised by hierarchical classification of the lichen sample.

There was a clear distinction in the types of compounds predicted to be produced by the BGCs between the two kingdoms (Figure 3), a finding that corroborates an earlier study comparing the biosynthetic space of fungi and bacteria in general (Robey et al. 2021). There was a preponderance of terpene BGCs recovered from the bacterial contigs (Figure 3A). Terpene compounds have been recovered in abundance from several lichen species with diverse bioactivities (González et al. 1974; Maier et al. 2009; Wang et al. 2009; Cui et al. 2012; Wijeratne et al. 2012; Li et al. 2015; Ahmed et al. 2017; Olivier‐Jimenez et al. 2019; Elkhateeb and Daba 2020; Ren et al. 2023). Considering the wealth of terpenes isolated from lichens, but absent a definitive link to any fungal BGCs (Singh 2023), we posit that some lichen‐derived terpenes may be of bacterial origin. There is relatively little known about the biosynthetic potential of lichen‐associated bacteria (Calcott et al. 2018), beyond cyanobionts (Kaasalainen et al. 2012; Ivanov et al. 2021), and culturable actinomycetes (Parrot et al. 2015, 2016; Liu et al. 2017; Sánchez‐Hidalgo et al. 2018; Hei et al. 2021; Hou et al. 2023). Indeed, a report from 2023 claims to be the first to report BGCs from entire lichen holobionts from two *Hypogymnia* species (Ahmad et al. 2023). It is thus challenging to place our findings within the context of other studies of the biosynthetic potential of lichen‐associated bacteria from a variety of lichen taxa. However, the high prevalence of terpene BGCs is a common feature of bacteria (Yamada et al. 2015; Reddy et al. 2020; Rudolf et al. 2021), as a high abundance of terpene BGCs has been reported for bacteria in other holobiont systems such as hypersaline stromatolites (Yi et al. 2024) and marine sponges (Loureiro et al. 2022; Wei et al. 2023; Nowak et al. 2024), but also in non‐holobiont environments, such as soil (Sharrar et al. 2020; Waschulin et al. 2022; Andreani‐Gerard et al. 2024), sediment (Suárez‐Moo and Prieto‐Davó 2024), and seawater (Paoli et al. 2022) habitats. Experiments investigating the effects of terpenes on the microbial communities of sorghum rhizobiomes found that the terpenes appeared to exert a regulatory effect on both bacteria and fungi, through inhibition of some populations and stimulation of others (Chou et al. 2023). Terpenes from bacteria have similarly been shown in other experiments to have inhibitory activity against a variety of microbes (Rudolf et al. 2021; Molina‐Aulestia et al. 2023). It is thus possible that bacterial terpene BGCs, if expressed, may play a role in regulating microbial populations in the lichen holobiont.

The fungal contigs, in contrast, contained a wealth of T1PKS BGCs (Figure 3B). The abundance of these BGCs has been readily documented (Bertrand and Sorensen 2018) and has given rise to a robust “PKS phylogeny” (Singh et al. 2022a). Type 1 PKS BGCs have been predicted to produce many lichen‐derived metabolites including orsellinic acid derivatives, such as gyrophoric acid (Singh et al. 2022a), the red pigment cristazarin (Paguirigan et al. 2023), and methylated orsellinic acid derivatives, such as atranonin (Kim et al. 2021), grayanic acid (Armaleo et al. 2011), and usnic acid (Abdel‐Hameed et al. 2016; Pizarro et al. 2020; Egbert et al. 2022). New methodologies facilitating the heterologous expression of BGCs from lichenized fungi (Kealey et al. 2021; Kim et al. 2021) will enable the discovery of novel bioactive compounds from these fungal players.

Most bacterial BGCs were found on contigs classified within phyla *Pseudomonadota* (13,056 BGCs), *Acidobacteria* (4188 BGCs), and *Actinomycetes* (2672 BGCs) (Figure S6A). However, these phyla are also the most abundant bacteria in the lichen samples (Figure 1, Table 1), and therefore the BGC counts may be biased by bacterial abundance. Correcting for sample sequence depth (Figure S7A), we found that there was a relatively even distribution of BGCs across the bacterial orders, but that orders within the *Alphaproteobacteria*, *Cyanophyceae* classes, and the phylum *Chloroflexota* had the greatest average number of complete BGCs per megabase pair of sequence data.

Of the 22,713 BGCs recovered from fungal contigs, at the level of phylum, a total of 22,634 BGCs (99.7%) were found on *Ascomycota* contigs, 34 BGCs (0.1%) were from contigs classified as *Basidiomycota*, and 45 (0.2%) BGCs were on contigs that could not be classified further than kingdom *Fungi* (Figure S6B, Dataset 7). Once these counts were corrected for the total sequence depth of individual metagenomic samples (Figure S7B), it was apparent that several orders—*Acarosporales* (*Lecanoromycetes*), *Trapeliales* (*Lecanoromycetes*), and *Verrucariales* (*Eurotiomycetes*) were particularly rich in biosynthetic potential. It must be noted that there is a potential for BGCs to have been counted twice if they were duplicated in diploid fungi (Tripp et al. 2017). A recent survey of 1037 fungal genomes where 36,399 BGCs were recovered, the researchers found that *Eurotiomycetes* tended to have the greatest number of BGCs per fungal genome (Robey et al. 2021), validating the result of *Verrucariales* (*Eurotiomycetes*) being particularly biosynthetically rich lichenized fungi. Conversely, a separate study surveying the biosynthetic potential of non‐lichenized *Dothideomycetes* species found them to be relatively biosynthetically rich (Gluck‐Thaler et al. 2020), a finding that our analyses do not support for their lichenized counterparts.

### 2.5 Biosynthetic Diversity

To ascertain the diversity of the BGCs in lichens, we clustered all 52,772 complete and fragmented bacterial and fungal BGCs into gene cluster families (GCFs), along with reference MiBIG BGCs, using BiG‐SCAPE (Navarro‐Muñoz et al. 2020) at a maximum distance of 0.3 (Dataset 9). Following the exclusion of GCFs consisting exclusively of reference BGCs, there were 26,894 GCFs. The biosynthetic classes of these GCFs are summarised in Table 1 of Dataset 9. A total of 21,515 GCFs consisted of a single BGC (singletons). The 5379 non‐singleton GCFs were split into 2619 exclusively fungal GCFs and 2760 exclusively bacterial GCFs (Dataset 9).

**FIGURE 4:**
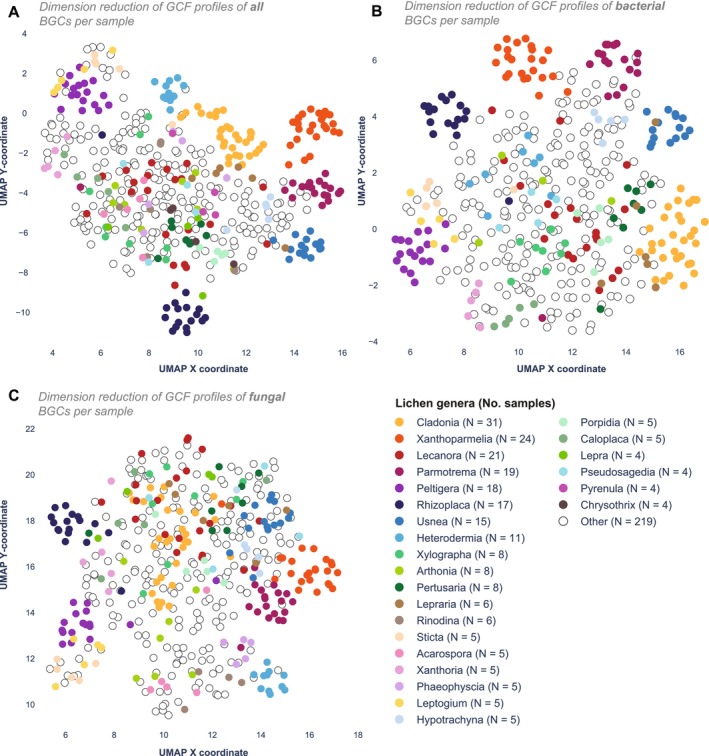
Dimension reduction via UMAP of GCF distributions across all lichen samples. (A) Dimension reduction of GCF profiles of all samples is suggestive of genus‐specific biosynthetic potential. (B) Dimension reduction of bacterial GCFs. (C) Dimension reduction of fungal GCFs. Lichen holobiont genera are indicated by colour, and the number of samples per genus is indicated in the legend. The axes correspond to coordinates in the reduced dimensional space and do not have inherent interpretability.

Applying UMAP (McInnes et al. 2018) dimension reduction to biosynthetic profiles revealed distinct clustering by lichen genera, indicating that lichen genera tend to share similar biosynthetic characteristics (Figure 4A). To confirm this observation, we considered only genera with four or more representatives in an analysis of similarity (ANOSIM) analysis, with a significance cutoff of *p* < 0.05 (Dataset 10). Results indicated that all lichen genera, except *Xylographa*, *Lepra*, *Pseudosagedia*, *Pyrenula*, *Chrysothrix*, *Sticta*, *Leptogium*, and *Arthonia*, had significantly different biosynthetic profiles from all other genera (Dataset 10). To discern whether the observed preservation of biosynthetic capacity across these genera stemmed from fungal or bacterial GCF profiles, we repeated the analyses with only bacterial GCFs and only fungal GCFs: ANOSIM indicated significant differences in bacterial GCF profiles across all examined lichen genera (Dataset 10). In contrast, fungal GCF profiles exhibited less differentiation, with only 52% of the lichen genera displaying distinct profiles (Dataset 10). These findings suggest that the biosynthetic diversity of lichen‐associated bacteria is influenced by the host lichen genus, with each genus harbouring a unique bacterial biosynthetic repertoire (Figure 4B, Dataset 10). This could be a reflection of lichen genera recruiting particular bacterial symbionts, or certain bacterial taxa being more fit for holobiont lifestyles with particular lichen genera, and subsequent conservation of lichen‐host‐specific bacterial populations (Grube et al. 2009; Bates et al. 2011), akin to that seen in several marine sponges (Schmitt et al. 2012). The fungal GCFs appear to follow a similar trend of species specificity but that the GCF distribution across taxonomic lineages is broader (Figure 4C) implying the potential prevalence of conserved biosynthetic pathways across genera for addressing shared ecological challenges. Similar trends were observed when researchers surveyed the biosynthetic potential in more than 1000 fungal genomes, where they found many fungal GCFs followed taxonomic lineages and were largely genus‐ or species‐specific, but that several GCFs spanned several subphyla (Robey et al. 2021). It must be noted, however, that the limited phylogenetic diversity of the lichenized fungi in our study, encompassing a single phylum (467 unique species), contrasts with the bacteria detected in our study, which span at least 101 phyla and thousands of species, and the greater BGC diversity in bacteria may be a consequence of their wider phylogenetic range. Additionally, a further limitation that must be considered is the bias in sampling: A large proportion of the metagenomic sequence datasets used in this study come from North American countries and from lichen within the *Lecanorales* order (Figure S1, Dataset 1). While the trend may hold true for the global distribution of lichens, the observed shared distribution of BGCs in lichenized fungi may be a result of data at hand being skewed by this combination of geographic and taxonomic similarity.

Consequently, our findings suggest that although both bacterial and fungal components of lichens possess substantial biosynthetic capabilities, focusing on diverse lichen genera is crucial for researchers seeking novel compounds from lichen‐associated bacteria. Investigating bacteria from different lichen species within the same genus is likely to yield similar biosynthetic profiles and, thus, possibly lead to repeated compound isolations.

Next, we aimed to assess which GCFs, and by extension BGCs, are consistently present across multiple lichen species. We determined that 24 GCFs (Figure S8) were present in at least 5% (40 samples) of all lichen holobionts included in this study. The most ubiquitous fungal GCFs were Terpene_32736 (distributed across 130 samples), Terpene_36860 (113 samples), and PKSI_35802 (84 samples). The most ubiquitous bacterial GCFs were Terpene_3485 (72 samples), Terpene_6049 (66 samples), and NRPS_14821 (66 samples) (Dataset 9). The consistent presence of only three bacterial GCFs underscores the idea that while lichen‐associated bacteria appear to have diverse biosynthetic abilities, their contribution to lichen biosynthesis is possibly tailored to specific lichen genera.

We then aimed to better characterize the three most conserved GCFs. First, we considered the two GCFs that were most ubiquitous across all lichens where both were classified as potentially encoding terpenes. There were 133 BGCs in Terpene_32736, which spanned 62 lichen genera, and 114 BGCs in Terpene_36860 spanning 57 lichen genera. Curiously, lichen holobionts with Terpene_32736 did not have BGCs from Terpene_36860, and vice versa (Figure S8). Closer inspection of the BGCs in these two GCFs revealed the consistent presence of only two genes in all BGCs, which were predicted to encode farnesyl‐diphosphate farnesyltransferase (squalene synthase) and DnaJ (Hsp40). Squalene synthase generates squalene via a head‐to‐head condensation of two molecules of farnesyl diphosphate (van der Donk 2015; Song et al. 2020). Squalene is a valuable intermediate terpene for the production of bioactive triterpenoids, such as sterols and hopanoids (Ghimire et al. 2016; Paramasivan and Mutturi 2022). Due to the variety of neighbouring genes present in the 247 BGCs of these two GCFs, the end products of these BGCs are likely not common but all share squalene as a precursor moiety. The conservation of two GCFs involved in squalene production in lichen has been independently identified in a different study (Singh et al. 2024), which suggests that these terpenes are potentially important to lichenized fungi. The BGCs of the third most ubiquitous GCF, PKSI_35802, shared a core PKS gene that showed the greatest similarity with the core PKS gene present in reference BGCs: BGC0000057 and BGC0002596 which encode “F9775A/F9775B/orsellinic acid” and “lecanoric acid/orsellinic acid,” respectively. Orsellinic acid and its derivatives are the most commonly isolated compounds from lichen species (Ranković and Kosanić 2021; Roy and Soni 2021; Gill et al. 2023), and therefore the recovery of this GCF is not unexpected.

**FIGURE 5:**
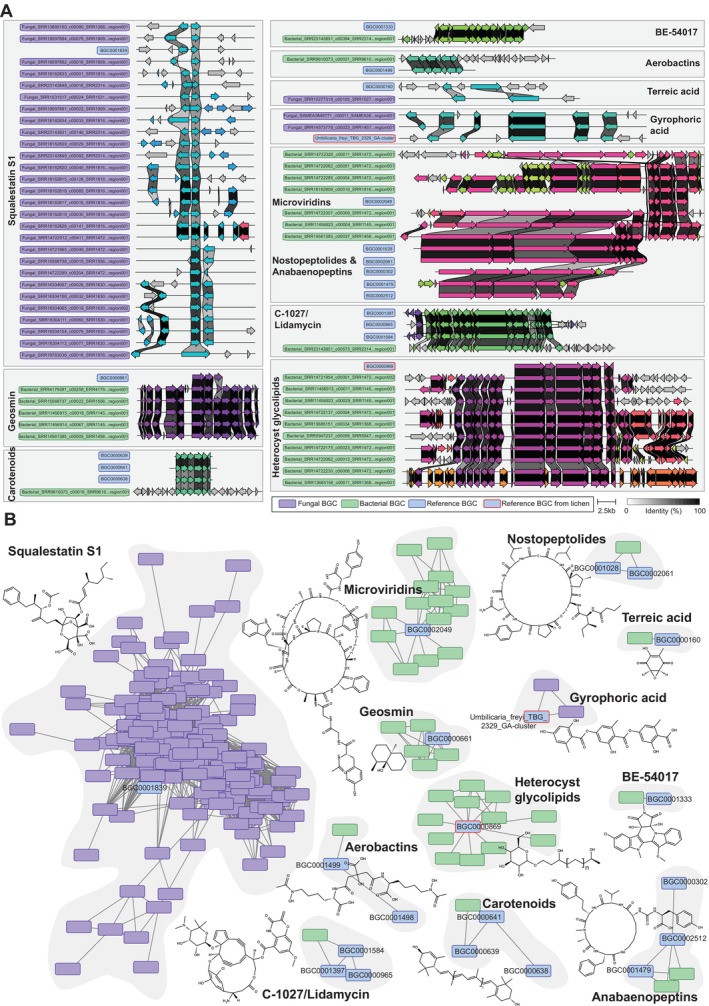
Complete BGCs recovered from lichens that share sequence homology and gene organisation with known reference biosynthetic gene clusters. (A) Alignment of complete BGCs per GCF that included a reference gene cluster. GCFs were identified in BiG‐SCAPE with a distance cutoff of 0.3, and visualised with clinker. Amino‐acid sequence identity between genes is indicated with a coloured scale. A scale bar is provided for gene size. The predicted origin of the BGCs (bacterial, fungal, and reference) is indicated with colour. (B) Network visualisation produced by BiG‐SCAPE of clusters that include known reference BGCs.

To account for fragmentation potentially leading to an overestimation of the BGC novelty, the fragmented BGCs were removed from the dataset and the BiG‐SCAPE analysis was repeated. The 7751 complete BGCs (3486 bacterial, 4265 fungal) were clustered into 4656 GCFs where 3747 (80.5%) of them were singletons, indicating that the majority of recovered complete BGCs were unique and reflected a high level of BGC diversity. The lack of clustering between these singleton BGCs and known BGCs suggests a degree of novelty, indicating potential for discovering new and interesting chemical compounds or drug candidates worthy of exploration. Of the remaining 909 GCFs, a total of 12 GCFs included reference BGCs (Figure 5A). The largest of these consisted exclusively of fungal‐derived BGCs and included the reference BGC for squalestatin S1 (Figure 5B). This GCF matches Terpene_32736 and Terpene_36860 from the previous analysis, likely sharing only a squalene precursor and not encoding squalestatin S1.

The next largest GCF consisted of 10 bacterial BGCs clustered with a BGC encoding heterocyst glycolipids (Figure 5) which was initially discovered in the *Nostoc* cyanobacterial symbiont of the lichen *Peltigera membranacea* (Kampa et al. 2013). Eight of the ten BGCs were predicted to originate from *Nostoc* species, and the other two from *Rhizonema*, with all host lichens belonging to the order *Peltigerales* (Dataset 7). In a similar vein, there were seven BGCs clustered with reference BGCs encoding microviridins, nostopeptolides, and anabaenopeptins, which are typically associated with cyanobacteria (Hoffmann et al. 2003; Liaimer et al. 2015; do Amaral et al. 2021; Monteiro et al. 2021; Zervou et al. 2021; Wang et al. 2022) but have been identified in other bacteria (Wang et al. 2022). These BGCs were recovered from contigs predicted to originate from *Nostoc* genera and were all hosted in lichen of the order *Peltigerales* (Dataset 7). Microviridins, nostopeptolides, and anabaenopeptins have all exhibited intriguing bioactivity, some of which could be exploited in a clinical setting (Ziemert et al. 2010; Liu et al. 2014; Schreuder et al. 2016; Zhang et al. 2018; Sieber et al. 2020; do Amaral et al. 2021; Wang et al. 2022).

Finally, a single bacterial BGC, predicted to originate from a *Streptomyces* sp., hosted within the critically endangered *Teloschistes peruensis* lichen (Dataset 7), shared sequence and organisational homology with the lidamycin gene cluster. Lidamycin is an enediyne compound with potent antitumor activity with IC_50_ values as low as 560 pM against the mouse myeloma SP2/0 cell line (Zhen et al. 2009), and 23 pM against the human lung carcinoma A549 cell line (Li et al. 2018). Prodrugs of the compound have since been developed that show increased anti‐tumour activity while decreasing cytotoxicity (Hong et al. 2024). This compelling example of bioactive compound recovery from endangered *Teloschistes peruensis* highlights the rich biosynthetic reservoir harboured by bacteria residing within lichen holobionts. However, it is crucial to acknowledge not only the endangered status of any given species but also the inherent limitations of relying on lichen biomass. Their slow growth and often recalcitrant nature in axenic culture underscore the importance of exploring alternative strategies. Therefore, we propose that culturing bacteria from small lichen samples represents a more sustainable and ethically responsible method for bioprospecting pharmaceutical compounds from these valuable holobionts.

We then wanted to survey the chemical diversity associated with lichen holobionts. To achieve this, we generated a pairwise Tanimoto similarity matrix, with a cutoff of 0.8 (80%) similarity, for all compounds cited in the literature as having been isolated from lichen as documented in Lichendex (https://lichendex.wixsite.com/my‐site/genomic‐data), with additional manual entries from Scifinder and Reaxys (*N* = 1571, Dataset 11).

**FIGURE 6:**
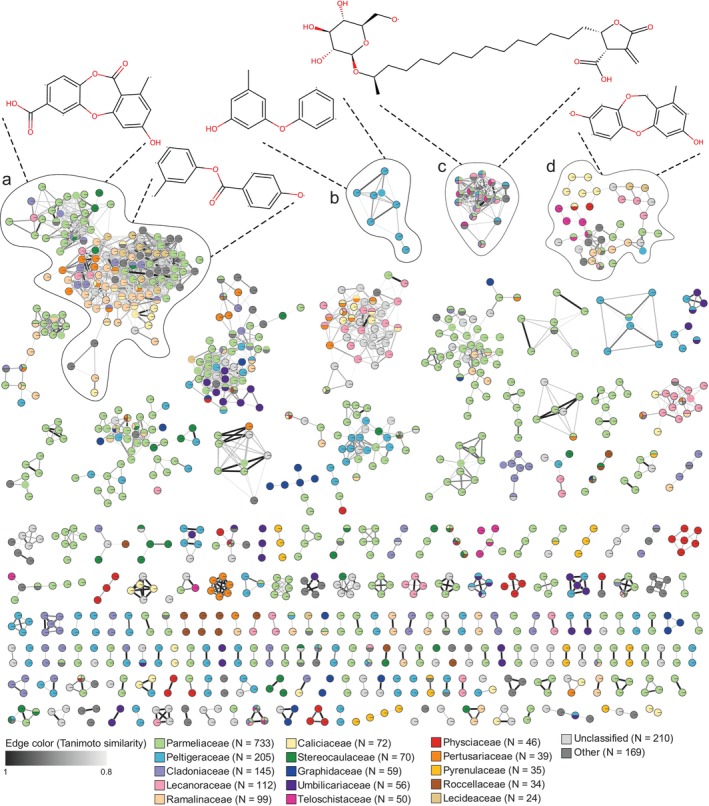
Structural similarity network of 1571 unique compounds, reported in the literature as recovered from lichen, based on Tanimoto similarity scores. The maximum common substructure is indicated for four clusters (a–d) illustrating different trends. Nodes represent individual compounds and are represented as pie charts showing the lichen families in which the compound is present. Similarities (edges) less than 0.8 are not included.

The chemical and metagenomic data are derived from different samples and sources; therefore, no links or inferences can be made between these two datasets. However, the survey of the known chemical space of lichen metabolites revealed that several scaffolds have been found in two or more lichen taxonomic families (Figure 6). For a high‐resolution image of the network with structures and names included with nodes, please see Figure S9. This trend of shared chemical scaffolds has similarly been observed in a recent study, wherein it was found that several compounds were shared between three different lichen genera, as determined by high resolution MS/MS data (Bracegirdle et al. 2025). The maximum common substructure for clusters of interest (Figure 6A—D) was generated using the ChemViz plugin (https://www.cgl.ucsf.edu/cytoscape/chemViz/) in CytoScape (Shannon et al. 2003). The largest cluster (Figure 6A) appeared to consist of two subclusters. The smaller subcluster primarily contained depsidone compounds featuring a dibenzodioxepin moiety. In contrast, the larger subcluster contained depsides. This suggests that the main difference between the subclusters is the presence or absence of an ether linkage. The pharmaceutical potential of these lichen‐derived compounds has been readily documented, with inhibitory activity in a variety of cancer cell lines (Nugraha et al. 2020; Thakur et al. 2023; Ureña‐Vacas et al. 2023; Bauri et al. 2024; Saha et al. 2024).

A common theme among clusters is for compounds to have been exclusively recovered from a single lichen family. An example of this is a group of eight phenolic ether‐containing compounds isolated solely from members of the *Peltigeraceae* family (Figure 6B). This may be a reflection of compounds that are produced exclusively by this particular lichen family, or that these compounds are produced in other families that have not yet been investigated or reported in the literature. Conversely, some compound clusters indicated wide distribution across several families, such as the murolic acid derivatives found in six known lichen families (Figure 6C). The prevalence of similar compounds across diverse families suggests these compounds may provide the lichens with solutions to shared challenges (Firn and Jones 2003; Wink 2003; Singh et al. 2024), such as antimicrobial defence or chemically maintaining the population structure of a holobiont community (Kampa et al. 2013). This compound ubiquity could indicate intriguing biological activities of interest to both ecologists and natural product chemists. Finally, we were intrigued to note a cluster of chlorinated depsidone compounds which included compounds such as diploicin, norvicanicin, and physciosporin (Figure 6D). The cluster consists of 38 compounds, of which only 5 were recovered from more than one family. Lichen‐derived chlorinated depsidones, such as physciosporin, exhibit anticancer activity in various cell lines (Yang et al. 2015, 2019; Taş et al. 2021), providing further evidence of lichens as a resource for novel anticancer pharmaceuticals.

The evidence presented here indicates that there is significant biosynthetic potential residing in lichen holobionts. However, we would be remiss if we did not point out that “potential” is a key word and that the presence of a given BGC does not imply constitutive or easily inducible production of the encoded compound. Indeed, the imbalance of biosynthetic potential and biosynthetic expression is a major hurdle to be overcome in natural product discovery research as researchers constantly uncover a dearth of “cryptic,” “silent,” or “orphan” BGCs (Hoskisson and Seipke 2020; Covington et al. 2021; Scherlach and Hertweck 2021). With this report, we do not mean to imply that the extensive collection of lichen tissue will inherently lead to the immediate discovery of numerous novel compounds. Rather, our objective is to underscore the substantial biosynthetic potential residing within lichen, particularly within their associated bacterial communities and propose that a subset of these microorganisms may be amenable to cultivation and genetic manipulation, offering a pathway towards the production of potentially bioactive molecules.

In conclusion, in our comprehensive survey encompassing 794 lichens from 34 countries, representing 467 unique lichen species, we unveiled the lichen holobiont's remarkable complexity. Our analysis suggests that the bacterial constituents of lichen holobionts possess a greater diversity of biosynthetic potential relative to their fungal counterparts, with indications of adaptation to their host genus. Our findings underscore the immense biosynthetic diversity and novelty harboured within lichen holobionts. While traditional approaches relying on direct isolation have faced limitations in uncovering the bioactive potential of lichens, the abundance of novel biosynthetic gene clusters within these holobionts presents a compelling opportunity for discovery. By leveraging existing and innovative molecular biology techniques, researchers could identify a wealth of bioactive compounds, reaffirming the significance of lichens as a promising frontier for drug discovery.

## 3 Materials and Methods

All scripts reported in the methods section can be found in the GitHub repository: https://github.com/samche42/Lichens/.

### 3.1 Metagenomic Assembly

**FIGURE 7:**
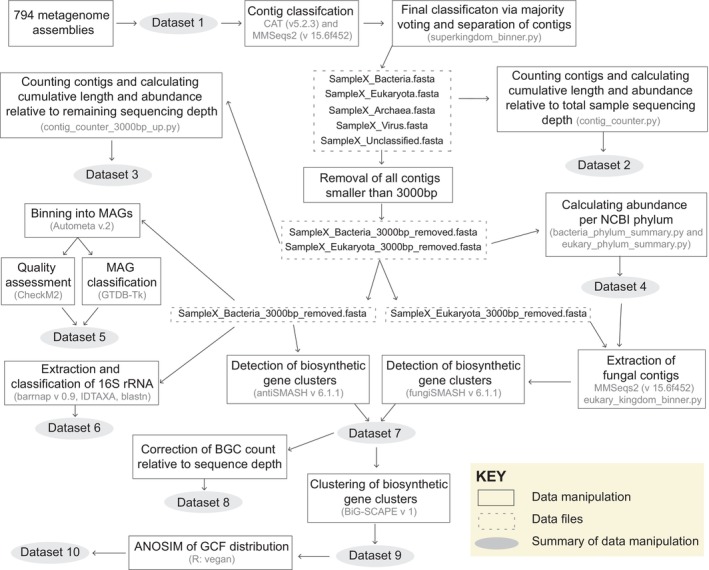
A summary of analyses performed in this study. Descriptions of analyses are shown within solid boxes. Resultant files, where pertinent, are boxed by dashed lines. Resultant datasets are denoted with grey ovals.

A list of 1189 raw data NCBI SRA (unassembled reads) accessions for various lichen species was sourced from Lichendex (https://lichendex.wixsite.com/my‐site/genomic‐data) and filtered down to a total of 963 datasets after metatranscriptomic and amplicon datasets were removed. Lichendex is available online as a rich resource for lichenologists, but the manuscript thoroughly describing the resource is still in the preparation stages. In short, the database is a manually curated collection of lists of assembled fungal genomes and lists of raw reads from NCBI, and molecules from a variety of sources including GNPS (Olivier‐Jimenez et al. 2019), NP Atlas (van Santen et al. 2019, 2022; Poynton et al. 2025), ChemBL (https://www.ebi.ac.uk/chembl/), PubChem (https://pubchem.ncbi.nlm.nih.gov/), and LOTUS (https://lotus.naturalproducts.net/). To uniformize the data and retrieve associated metadata for the datasets included in this study, custom scripts (Prepping_data_list.ipynb) were used, which deployed entrez (Schuler et al. 1996; Baxevanis 2006) functions “esearch” and “efetch” (Dataset 1). Sequence data were retrieved for all Illumina and IonTorrent dataset accessions using fastq‐dump (v. 3.0.8) from sra‐tools (Sayers et al. 2022). Long read datasets were manually downloaded from the NCBI web interface. Adapters from Illumina data were trimmed using trimmomatic (0.39) (Bolger et al. 2014) using the TruSeq3‐PE adapter library as reference and reads assembled using SPAdes (v.3.15.5) (Bankevich et al. 2012) with default settings. We were made aware during review that some sequence datasets may have been generated using Nextera adapters, and as such, these adapters would not have been removed and may have compromised the assembly of some datasets. For all assemblies, metrics for assembly size, number of contigs, number of contigs > 3000 bp, N50, and longest contig were calculated using assembly_stats.py. All assemblies with less than 1000 contigs greater than 3000 bp in size and an N50 smaller than 10,000 (*N* = 169) were removed from the dataset. This resulted in 794 metagenome assemblies as the final set for all downstream analyses. Due to the complexity of downstream analyses, we have provided a summarised workflow (Figure 7).

### 3.2 Taxonomic Classification of Contigs

Contigs were classified into superkingdom bins (*Bacteria*, *Archaea*, *Eukaryote*, *Virus*, and Unclassified) using the Contig Annotation Tool (CAT, v5.2.3) (von Meijenfeldt et al. 2019), and MMSeqs2 (v. 15.6f452) (Hauser et al. 2016; Steinegger and Söding 2017; Mirdita et al. 2021) with the NCBI database as reference. The final classification was determined via a majority voting approach using the superkingdom_binner.py. The number of contigs, cumulative length, and relative abundance for each superkingdom were calculated using contig_counter.py (Dataset 2). Contigs smaller than 3000 bp were then removed from all samples, and the calculations were repeated using contig_counter_3000bp_up.py (Dataset 3). Briefly, the estimated coverage of each contig was corrected for the length of the contig, and then the total coverage for the sample was calculated as the sum of all contigs' length corrected coverages. Relative abundance of the contig was then calculated as each contig's length‐corrected coverage divided by the total sample coverage. All downstream analyses were subsequently performed on datasets with contigs smaller than 3000 bp removed. The relative abundance of individual phyla from the bacterial and eukaryotic contigs was calculated using bacteria_phylum_summary.py and eukary_phylum_summary.py, respectively (Dataset 4). Here, the abundance per phylum was calculated using the relative abundance of the contig calculated in the previous step. Additionally, we classified only the bacterial contigs (> 3000 bp) using MMSeqs2 (v. 15.6f452) (Hauser et al. 2016; Steinegger and Söding 2017; Mirdita et al. 2021) with the GTDB dataset (v. 214.1) as a reference (Dataset 5). Classification using CAT was not possible due to a documented programmatic bug (https://github.com/MGXlab/CAT_pack/issues/123 and https://github.com/MGXlab/CAT_pack/issues/68). The GTDB does not include eukaryotic sequences, and therefore could not be used as a secondary classification reference for eukaryotic contigs.

### 3.3 Bacterial MAGs

Bacterial contigs greater than 3000 bp in length were clustered into MAGs using Autometa (v 2.2.1) (Miller et al. 2019; Rees et al. 2023). All recovered bins were assessed for quality using CheckM2 (v.1.0.2) (Chklovski et al. 2023) and taxonomically classified using GTDB‐Tk (v.2.3.2) (Chaumeil et al. 2019) using Release 214 as reference. Basic metrics such as genome size, N50, and GC content were calculated using binning_summary.py (Dataset 6).

### 3.4 Extraction and Analysis of Bacterial Ribosomal Gene Sequences

Ribosomal sequences were extracted from all scaffolds from all lichen metagenomes using barrnap (v. 0.9) (Seemann 2013). The 16S rRNA gene sequences were separated from the barrnap output files using ribosomal_extractor.py. The 16S rRNA gene sequences were classified using IDTAXA (Murali et al. 2018) at a confidence threshold of 60% against the modified GTDB 16S dataset (v. 214.1) (Dataset 7). The 16S rRNA sequences were also classified using the blastn (Camacho et al. 2009) tool database with the GTDB 16S rRNA dataset (v. 214.1) as a reference. Only the best hit returned for each 16S sequence and only matches with a sequence identity of > 95% were kept (Dataset 7).

### 3.5 Detection of BGCs

BGCs were predicted for all bacterial contigs greater than 3000 bp in length using antiSMASH (v 6.1.1) (Blin et al. 2021). Eukaryotic contigs were split into fungal (*Fungi*), plant (*Viridiplantae*), animals (*Metazoa*), and unclassified kingdom fasta files using eukary_kingdom_binner.py. This separation was based on the MMSeqs2 classification, as this has been shown to perform particularly well with eukaryotic sequences (Mirdita et al. 2021). BGCs were detected in fungal contigs (greater than 3000 bp) using antiSMASH (v 6.1.1) (Blin et al. 2021) with the—taxon parameter set to fungi and the—genefinding‐tool parameter set to glimmerhmm. A basic summary of all BGCs was generated for all BGCs using antismash_summary.py (Dataset 8).

### 3.6 Clustering of BGCs

All 52,772 identified bacterial and fungal BGCs were clustered into GCFs using BiG‐SCAPE (Navarro‐Muñoz et al. 2020), with a maximum distance cutoff of 0.3, with MIBiG (Terlouw et al. 2023) BGCs included, clans disabled, and mode set to auto (Dataset 8). We also included reference BGCs for gyrophoric acid (Singh et al. 2022a) and atranorin (Kim et al. 2021), which are not included in the MiBIG 3 release. While we acknowledge the potential to include more reference BGCs by increasing the GCF distance threshold, we initially used and subsequently opted to retain the default distance of 0.3 as we felt this gave an acceptable balance of sensitivity with specificity, that is, manual inspection of GCFs revealed similar BGCs being incorporated into a given GCF but that some shared at most two common genes. Increasing the maximum distance may overinflate potential similarities leading to erroneous assumptions about common BGCs and their predicted products. Additionally, the scale of the data and the computational time and resources required to run the analyses with more lenient cutoffs could not be justified given the predicted outcome. All BGCs are available on FigShare (please find links in Data Availability section) and researchers are encouraged to repeat the analysis with less stringent cutoffs if they wish to explore this space. The GCF distribution across samples was counted (see GCF_wrangling.ipynb) (Dataset 9). Importantly, if a lichen species was represented multiple times, an average count per GCF per species was calculated so as not to inflate similarities within a genus through the presence of pseudoreplicates. The resulting matrix was subjected to dimension reduction with UMAP (McInnes et al. 2018), with maximum neighbours set to 15 and the minimum distance set to 1, and reduced to both three‐ and two‐dimensional representations (please see GCF_dim_reduc.ipynb for full details and 3‐D rendering). Lichen samples with four or more representatives of a holobiont genus were subset out for ANOSIM analysis in R using the vegan (Dixon 2003) package, with the distance metric set to Bray‐Curtis, with 9999 permutations (Dataset 10). The complete BGCs were then subset out and the BiG‐SCAPE analysis repeated as before (Dataset 9). Clusters that included reference genomes were visualised in clinker (Gilchrist and Chooi 2021) and associated networks were visualised in CytoScape (Shannon et al. 2003).

### 3.7 Molecular Similarity Networking of Compounds Isolated From Lichen Holobionts

The majority of structural data for lichen‐derived compounds were downloaded from Lichendex (https://lichendex.wixsite.com/my‐site/blank‐2) as well as Scifinder and Reaxys. Entries were manually curated, and additional compounds were included, resulting in 1571 unique compounds (Dataset 11). The Tanimoto similarity was calculated for all pairwise combinations of the compounds using RDKit (Landrum 2013), using Morgan fingerprints generated with a radius of 2 and a bit vector 2048 bits long, where only similarities greater than or equal to 0.8 were kept. This matrix was visualised in CytoScape (Shannon et al. 2003) with the yFiles Organic layout. Maximum common substructure per cluster was generated using the ChemViz plugin (https://www.cgl.ucsf.edu/cytoscape/chemViz/) in CytoScape (Shannon et al. 2003).

## Author Contributions

**Samantha C. Waterworth:** conceptualization, data curation, formal analysis, investigation, methodology, software, visualization, writing – original draft, writing – review and editing. **Susan Egbert:** data curation, methodology, writing – review and editing. **John Sorensen:** funding acquisition, supervision, writing – review and editing. **Barry R. O'Keefe:** funding acquisition, supervision, writing – review and editing. **John A. Beutler:** funding acquisition, supervision, project administration, writing – review and editing.

## Conflicts of Interest

The authors declare no conflicts of interest.

## Supporting information


**FIGURE S1.** Collection sites of all lichen metagenome samples successfully assembled from publicly available data. Collection points are coloured by the taxonomic order of lichen and sized relative to the number of samples from each unique set of coordinates. The map was generated in Spotfire. (A) Global overview of collection sites of lichen holobionts included in this study. (B) Magnified view of samples collected in North America. (C) Magnified view of samples collected in Europe.

**FIGURE S2.** The cumulative length, number, and relative abundance of contigs in 794 lichen samples categorised by superkingdom before and after the removal of contigs smaller than 3000 bp in length. All lichen samples have been organised in a hierarchical manner in descending taxonomic order as indicated by the coloured legend.

**FIGURE S3.** Bacterial MAGS recovered from 794 lichen holobiont metagenomes. (A) The quality distribution of MAGs across all samples. (B) The taxonomic distribution of high and medium quality MAGs at the phylum level from all samples.

**FIGURE S4.** Survey of 16S rRNA gene sequences from 794 lichen holobiont metagenomes. (A) The length distribution of recovered 16S rRNA gene sequences from all samples. (B) The count distribution of the number of 16S sequences recovered per sample per sequencing technology.

**FIGURE S5.** The distribution of BGCs across lichen samples. (A) The raw count of recovered BGCs per lichen sample. (B) The BGC count per sample when corrected for total sequencing depth. (C) The BGC count per sample when corrected for the sequencing depth of all contigs greater than 3000 bp. For panels A—C, the predicted source and whether the BGCs are predicted to be complete or fragmented is indicated by a coloured key. (D) A comparison of the average number of BGCs from predicted fungal and bacterial sources per lichen genus.

**FIGURE S6.** The distribution of complete and fragmented biosynthetic gene clusters (BGCs) in lichen holobionts as found in (A) bacterial and (B) fungal contigs. The number, length distribution, and taxonomic phylogeny of complete and fragmented BGCs are provided.

**FIGURE S7.** The distribution of the average number of complete and fragmented biosynthetic gene clusters (BGCs) per Mbp of metagenomic data per lichen holobiont metagenome as found in (A) bacterial and (B) fungal contigs. The types of BGCs per taxonomic order are indicated with a coloured key.

**FIGURE S8.** Distribution of GCFs present in at least 5% (*N* = 40) of the 794 lichen holobiont samples. GCF presence is indicated by blue blocks. Samples are organised hierarchically by taxonomic lineage and indicated by colour. The number of samples in which the GCF is present is presented as a barplot on the right. The predicted source of BGCs within the GCF is indicated by colour.

**FIGURE S9.** Structural similarity network of 1571 unique compounds, reported in the literature as recovered from lichen, based on Tanimoto similarity scores. Nodes represent individual compounds and are represented as pie charts showing the lichen families in which the compound is present. Compound structures and names are included with each node.

**DATASET 1.** Details and summary of all datasets used in this study.

**DATASET 2.** Metrics for all contigs recovered per lichen metagenomic dataset.

**DATASET 3.** Metrics for contigs recovered per lichen metagenomic dataset after removal of all contigs smaller than 3000 bp.

**DATASET 4.** Summaries of bacterial and eukaryotic phyla abundance.

**DATASET 5.** Metrics and summaries of MAGs recovered from all lichen datasets.

**DATASET 6.** Summaries of recovered 16S rRNA gene sequences recovered from all lichen datasets.

**DATASET 7.** Details and summaries of all BGCs recovered from all lichen datasets.

**DATASET 8.** Summarized metrics of all BGCs from all lichen datasets when adjusted for sequencing depths.

**DATASET 9.** Summaries of BGC clustering into GCFs.

**DATASET 10.** ANOSIM results for GCF distribution in lichen genera.

**DATASET 11.** Summary of all compounds reported in lichen.

## Data Availability

All raw data used in this study were publicly available and accession numbers have been provided in Dataset 1. All recovered MAGs, BGCs, and associated BiG‐SCAPE analyses are available in FigShare at Project: https://figshare.com/projects/A_biosynthetic_and_taxonomic_atlas_of_the_global_lichen_holobiont/211204. Specifically, the BGCs can be accessed at DOI 10.6084/m9.figshare.26109124, the associated BiG‐SCAPE analyses can be accessed at DOI 10.6084/m9.figshare.26110078, and the bacterial MAGs can be accessed at DOI 10.6084/m9.figshare.26110162. All scripts used for data processing and visualisation are available on GitHub at https://github.com/samche42/Lichens.
