# Supplementary material for: A Biosynthetic and Taxonomic Atlas of the Global Lichen Holobiont
Source: Environ Microbiol. 2025 Jun 4;27(6):e70112. doi: 10.1111/1462-2920.70112 (PMC12136951; doi:10.1111/1462-2920.70112)
Supplement: Supplementary file 1 — FIGURE S1. Collection sites of all lichen metagenome samples successfully assembled from publicly available data. Collection points are coloured by the taxonomic order of lichen and sized relative to the number of samples from each unique set of coordinates. The map was generated in Spotfire. (A) Global overview of collection sites of lichen holobionts included in this study. (B) Magnified view of samples collected in North America. (C) Magnified view of samples collected in Europe. [file EMI-27-e70112-s013.pdf]

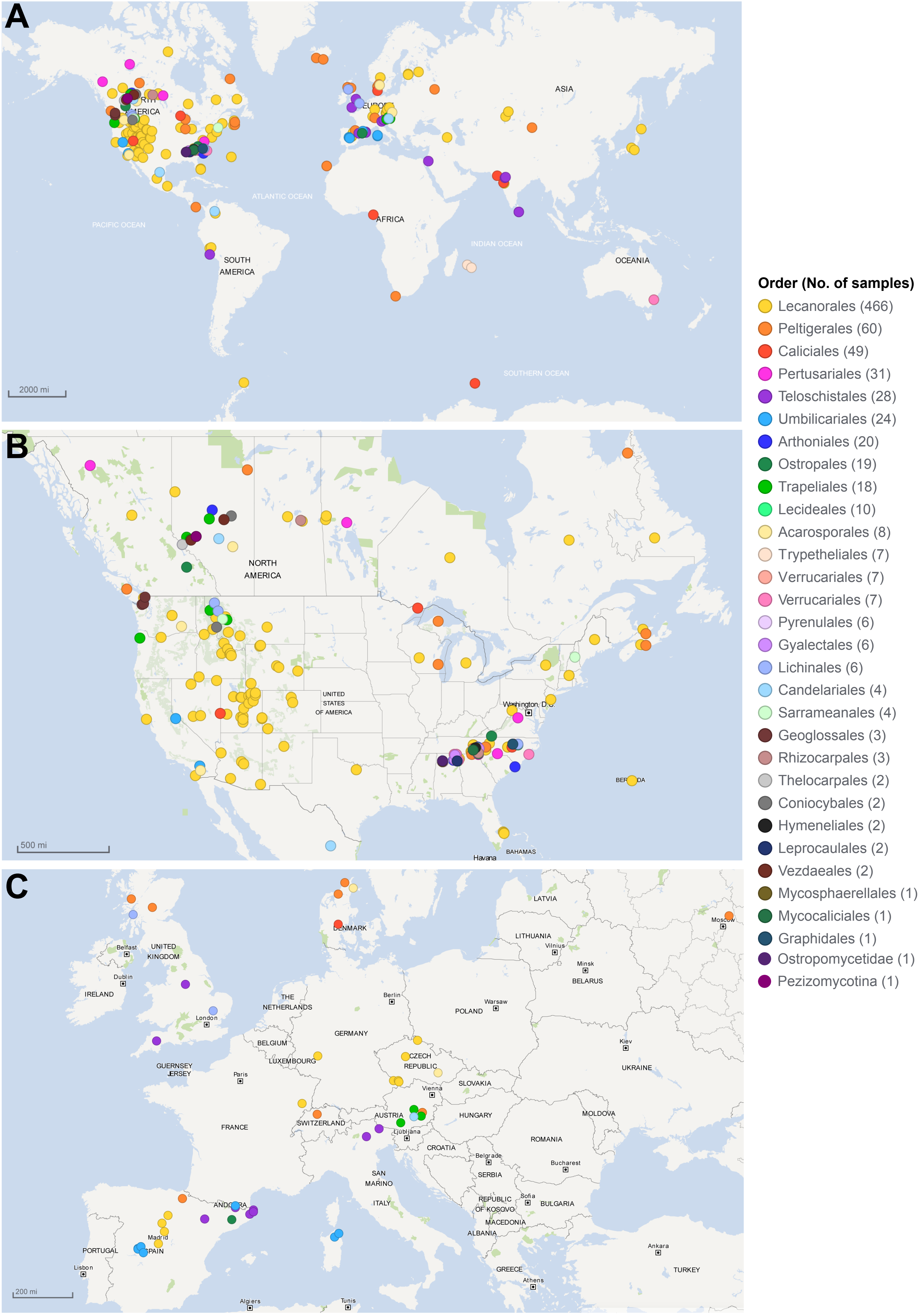
A
B
C
NORTH AMERICA
SOUTH AMERICA
EUROPE
ASIA
AFRICA
OCEANIA
ATLANTIC OCEAN
PACIFIC OCEAN
INDIAN OCEAN
SOUTHERN OCEAN
2000 mi
UNITED STATES OF AMERICA
Washington, D.C.
BAHAMAS
Havana
500 mi
UNITED KINGDOM
IRELAND
Belfast
Dublin
London
THE NETHERLANDS
BELGIUM
LUXEMBOURG
GERMANY
Berlin
POLAND
Warsaw
LATVIA
LITHUANIA
Vilnius
Minsk
BELARUS
Moscow
Kiev
UKRAINE
CZECH REPUBLIC
SLOVAKIA
Vienna
AUSTRIA
HUNGARY
SWITZERLAND
FRANCE
Paris
GUERNSEY
JERSEY
Ljubljana
CROATIA
ROMANIA
MOLDOVA
Bucharest
Belgrade
SERBIA
REPUBLIC OF KOSOVO
Sofia
BULGARIA
MACEDONIA
ALBANIA
SAN MARINO
ITALY
GREECE
Athens
TURKEY
Ankara
SPAIN
Madrid
PORTUGAL
Lisbon
Algiers
Tunis
200 mi
Order (No. of samples)
Lecanorales (466)
Peltigerales (60)
Caliciales (49)
Pertusariales (31)
Teloschistales (28)
Umbilicariales (24)
Arthoniales (20)
Ostropales (19)
Trapeliales (18)
Lecideales (10)
Acarosporales (8)
Trypetheliales (7)
Verrucariales (7)
Verrucariales (7)
Pyrenulales (6)
Gyalectales (6)
Lichinales (6)
Candelariales (4)
Sarrameanales (4)
Geoglossales (3)
Rhizocarpales (3)
Thelocarpales (2)
Coniocybales (2)
Hymeneliales (2)
Leprocaulales (2)
Vezdaeales (2)
Mycosphaerellales (1)
Mycocaliciales (1)
Graphidales (1)
Ostropomycetidae (1)
Pezizomycotina (1)
