# Supplementary figures and images for: A Biosynthetic and Taxonomic Atlas of the Global Lichen Holobiont
Source: Environ Microbiol. 2025 Jun 4;27(6):e70112. doi: 10.1111/1462-2920.70112 (PMC12136951; doi:10.1111/1462-2920.70112)

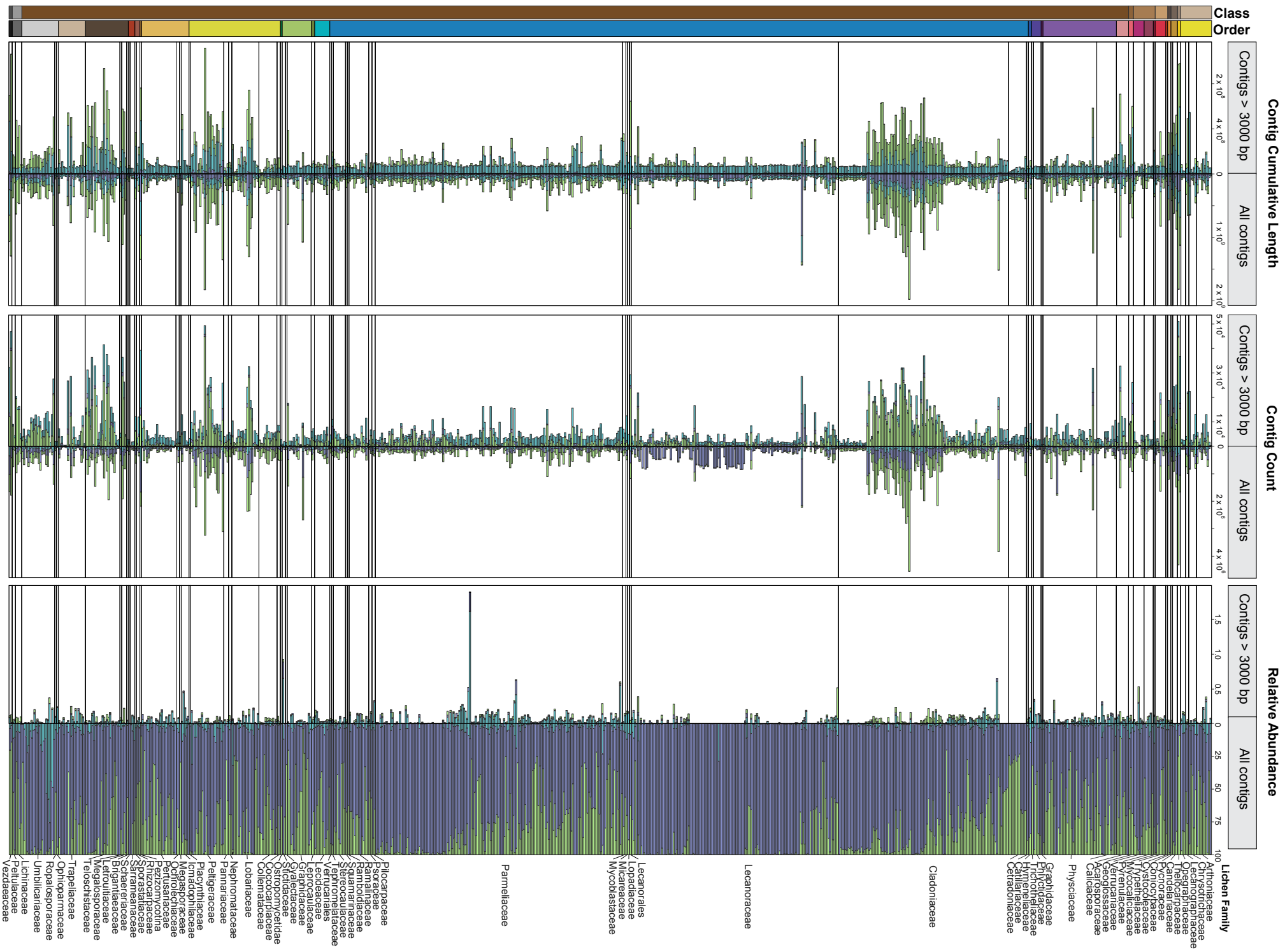

Supplement: Supplementary file 2 — FIGURE S2. The cumulative length, number, and relative abundance of contigs in 794 lichen samples categorised by superkingdom before and after the removal of contigs smaller than 3000 bp in length. All lichen samples have been organised in a hierarchical manner in descending taxonomic order as indicated by the coloured legend. [file EMI-27-e70112-s012.pdf]

**A**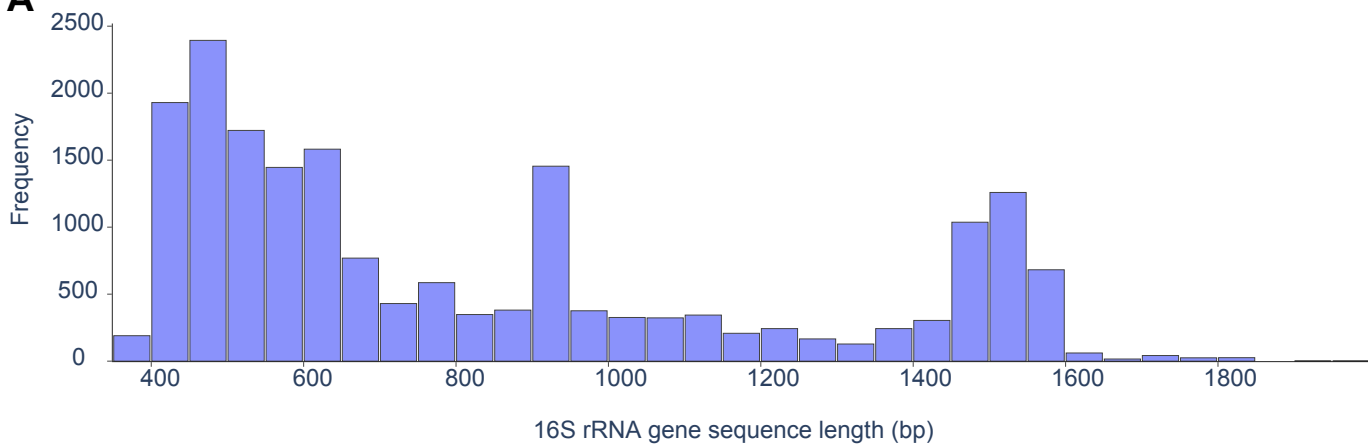**B**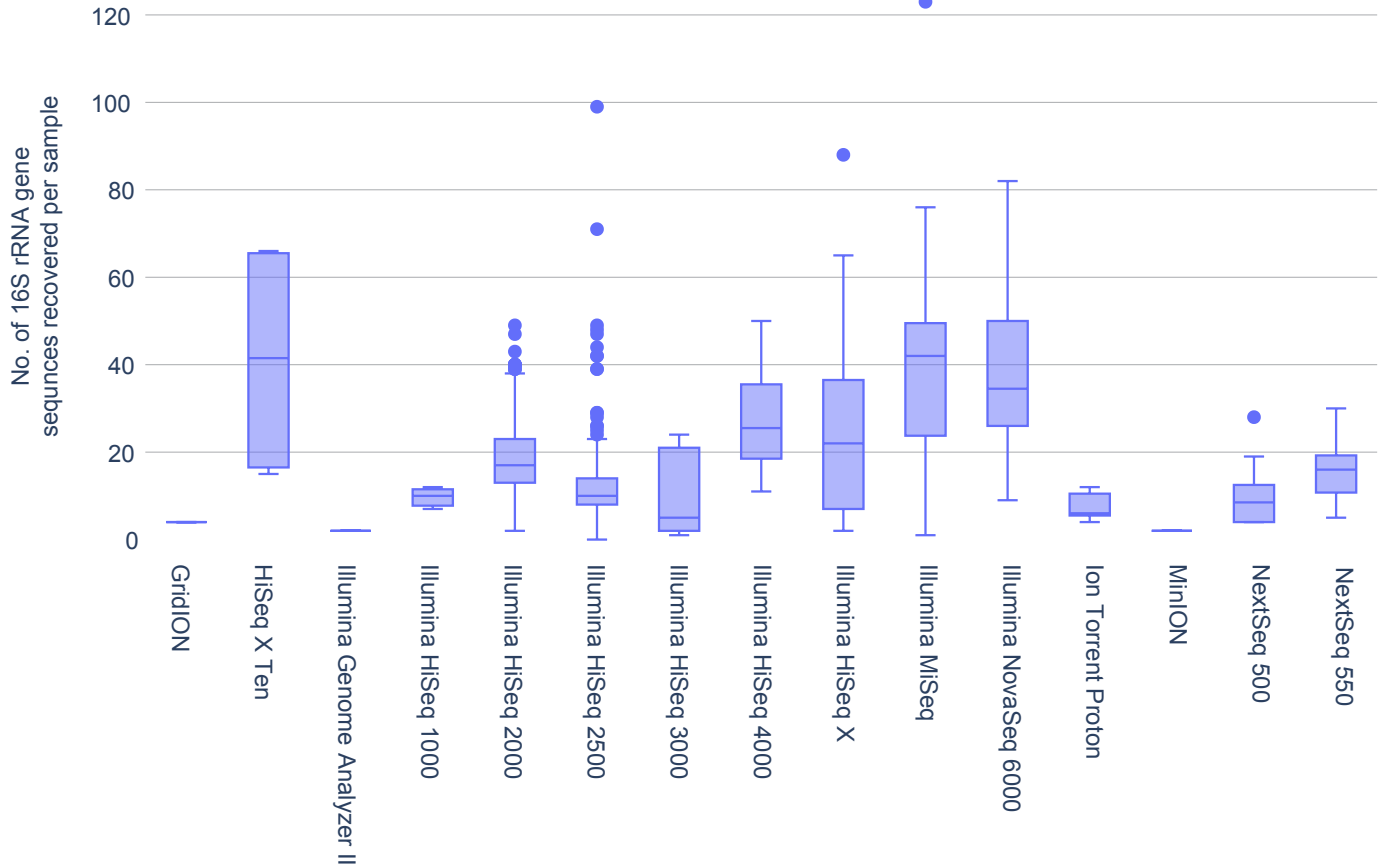

Supplement: Supplementary file 4 — FIGURE S4. Survey of 16S rRNA gene sequences from 794 lichen holobiont metagenomes. (A) The length distribution of recovered 16S rRNA gene sequences from all samples. (B) The count distribution of the number of 16S sequences recovered per sample per sequencing technology. [file EMI-27-e70112-s011.pdf]

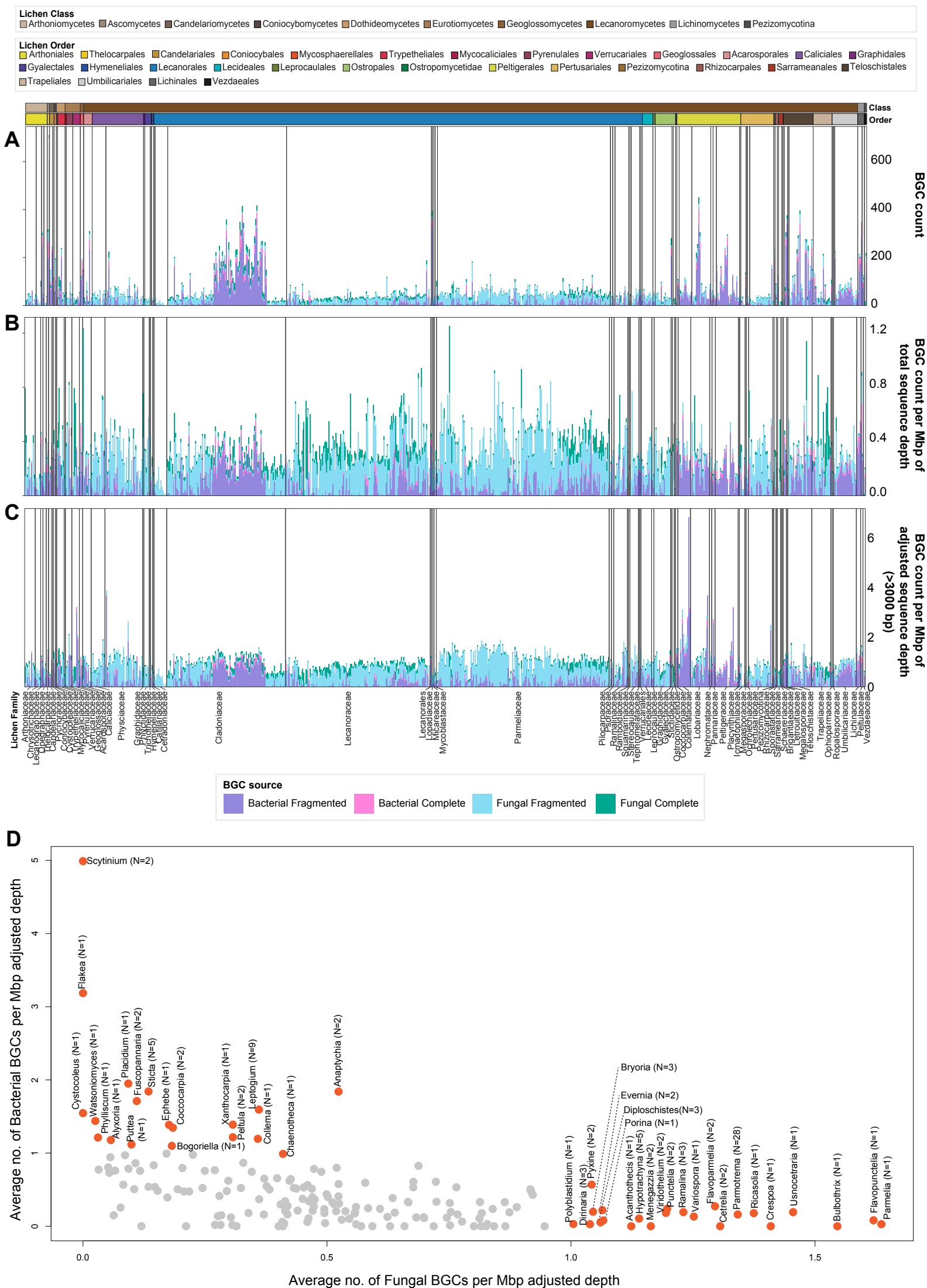

Supplement: Supplementary file 5 — FIGURE S5. The distribution of BGCs across lichen samples. (A) The raw count of recovered BGCs per lichen sample. (B) The BGC count per sample when corrected for total sequencing depth. (C) The BGC count per sample when corrected for the sequencing depth of all contigs greater than 3000 bp. For panels A—C, the predicted source and whether the BGCs are predicted to be complete or fragmented is indicated by a coloured key. (D) A comparison of the average number of BGCs from predicted fungal and bacterial sources per lichen genus. [file EMI-27-e70112-s007.pdf]

A

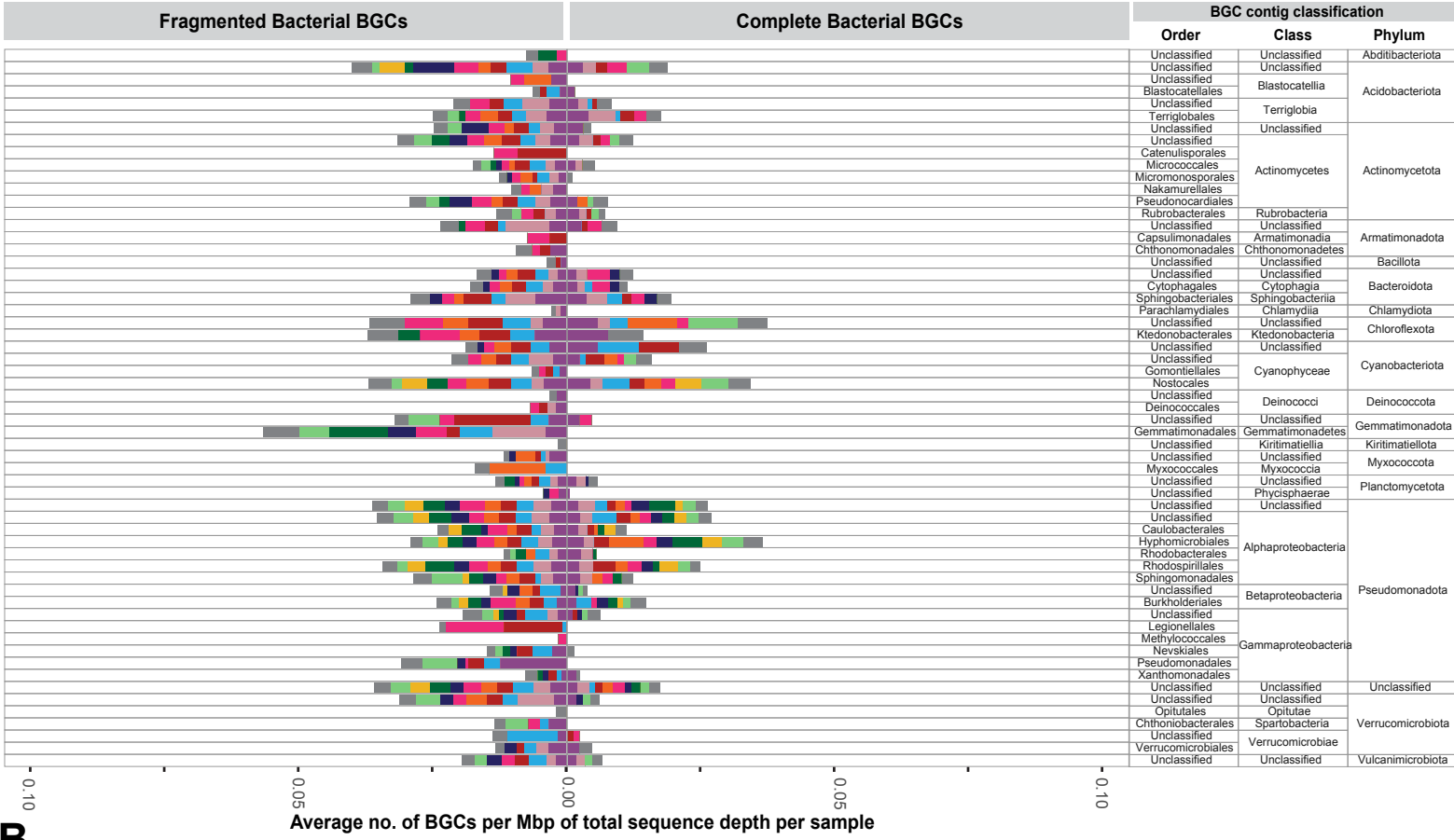

B

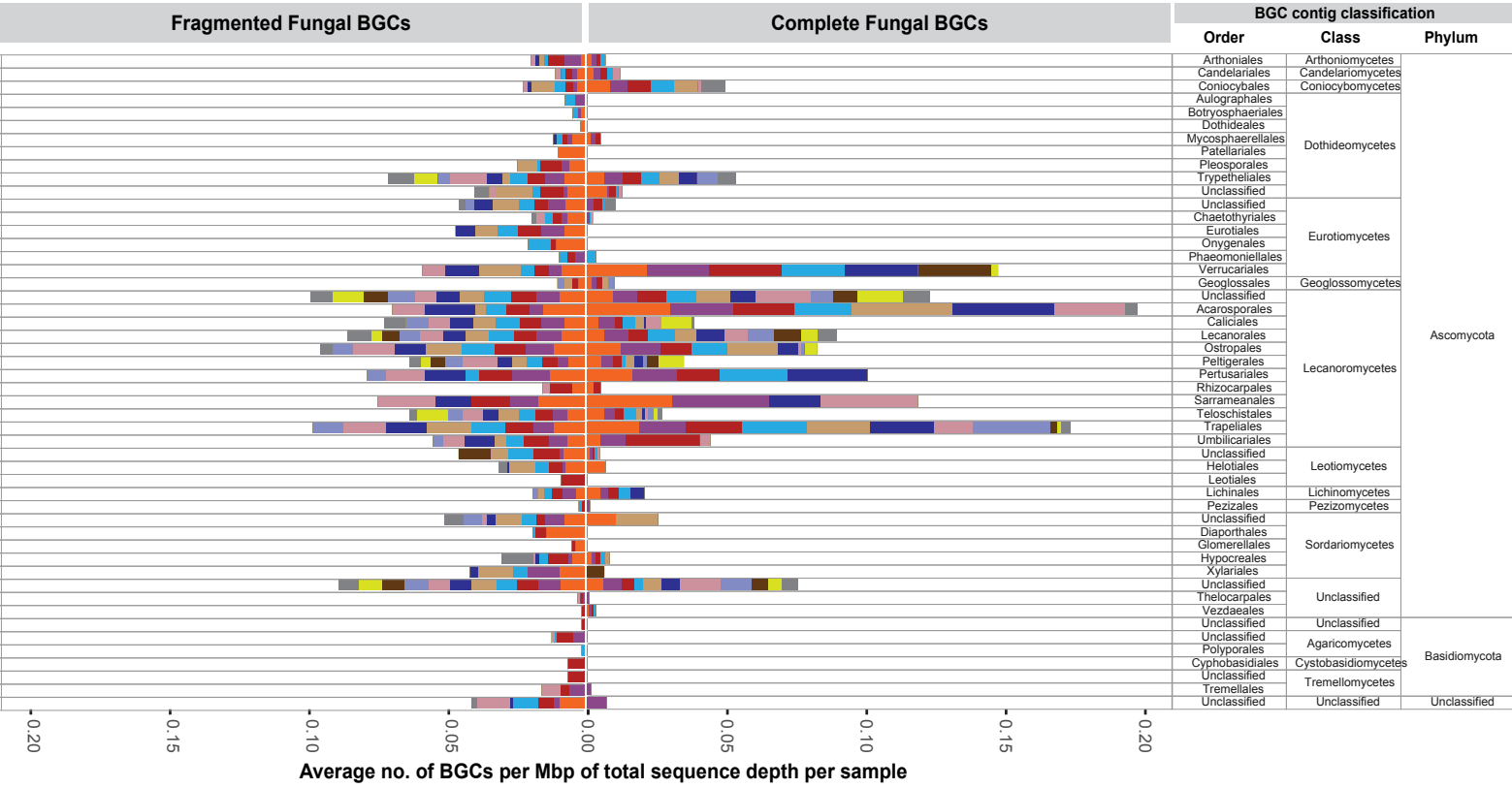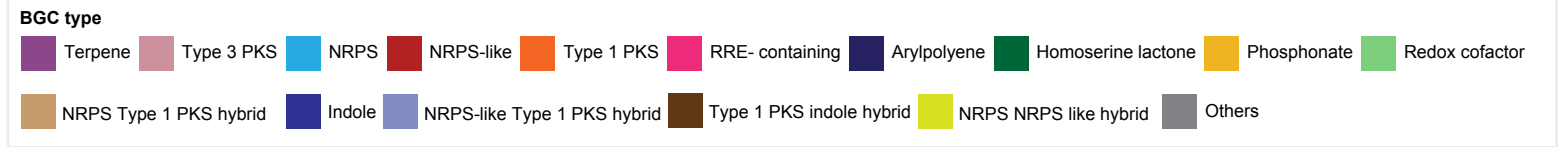

Supplement: Supplementary file 7 — FIGURE S7. The distribution of the average number of complete and fragmented biosynthetic gene clusters (BGCs) per Mbp of metagenomic data per lichen holobiont metagenome as found in (A) bacterial and (B) fungal contigs. The types of BGCs per taxonomic order are indicated with a coloured key. [file EMI-27-e70112-s020.pdf]

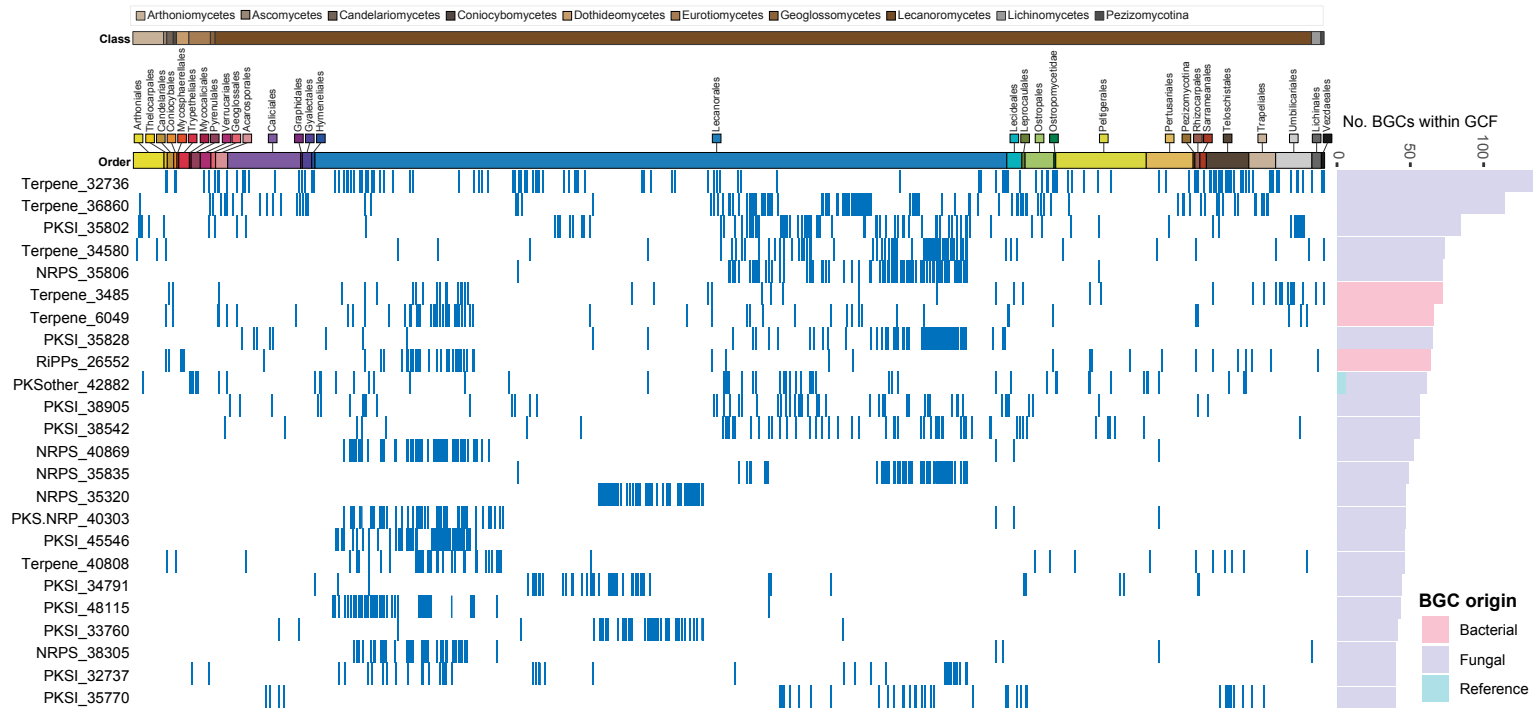

Supplement: Supplementary file 8 — FIGURE S8. Distribution of GCFs present in at least 5% (N = 40) of the 794 lichen holobiont samples. GCF presence is indicated by blue blocks. Samples are organised hierarchically by taxonomic lineage and indicated by colour. The number of samples in which the GCF is present is presented as a barplot on the right. The predicted source of BGCs within the GCF is indicated by colour. [file EMI-27-e70112-s004.pdf]

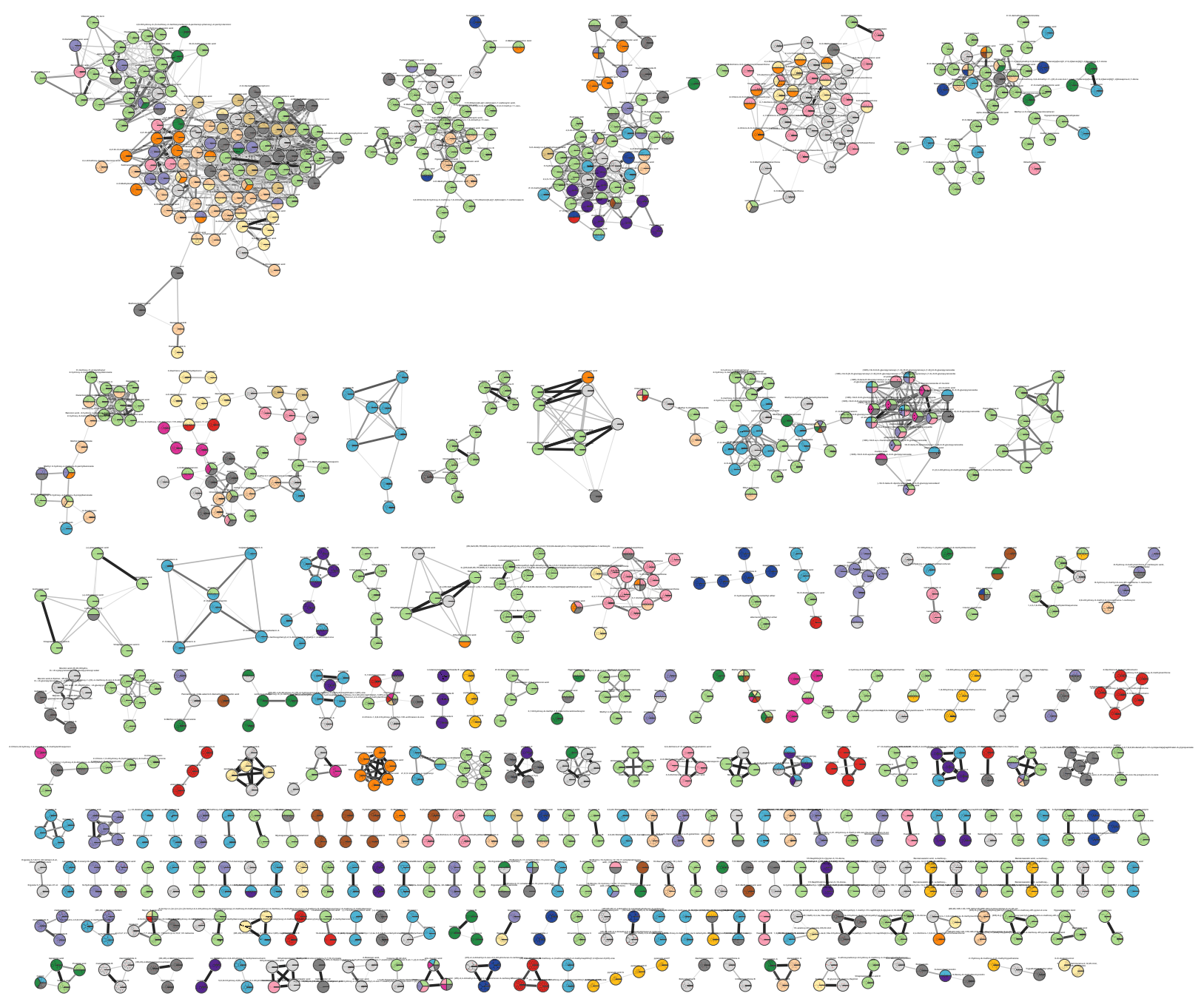

Supplement: Supplementary file 9 — FIGURE S9. Structural similarity network of 1571 unique compounds, reported in the literature as recovered from lichen, based on Tanimoto similarity scores. Nodes represent individual compounds and are represented as pie charts showing the lichen families in which the compound is present. Compound structures and names are included with each node. [file EMI-27-e70112-s015.pdf]
