# Supplementary material for: A Biosynthetic and Taxonomic Atlas of the Global Lichen Holobiont
Source: Environ Microbiol. 2025 Jun 4;27(6):e70112. doi: 10.1111/1462-2920.70112 (PMC12136951; doi:10.1111/1462-2920.70112)
Supplement: Supplementary file 6 — FIGURE S6. The distribution of complete and fragmented biosynthetic gene clusters (BGCs) in lichen holobionts as found in (A) bacterial and (B) fungal contigs. The number, length distribution, and taxonomic phylogeny of complete and fragmented BGCs are provided. [file EMI-27-e70112-s002.pdf]

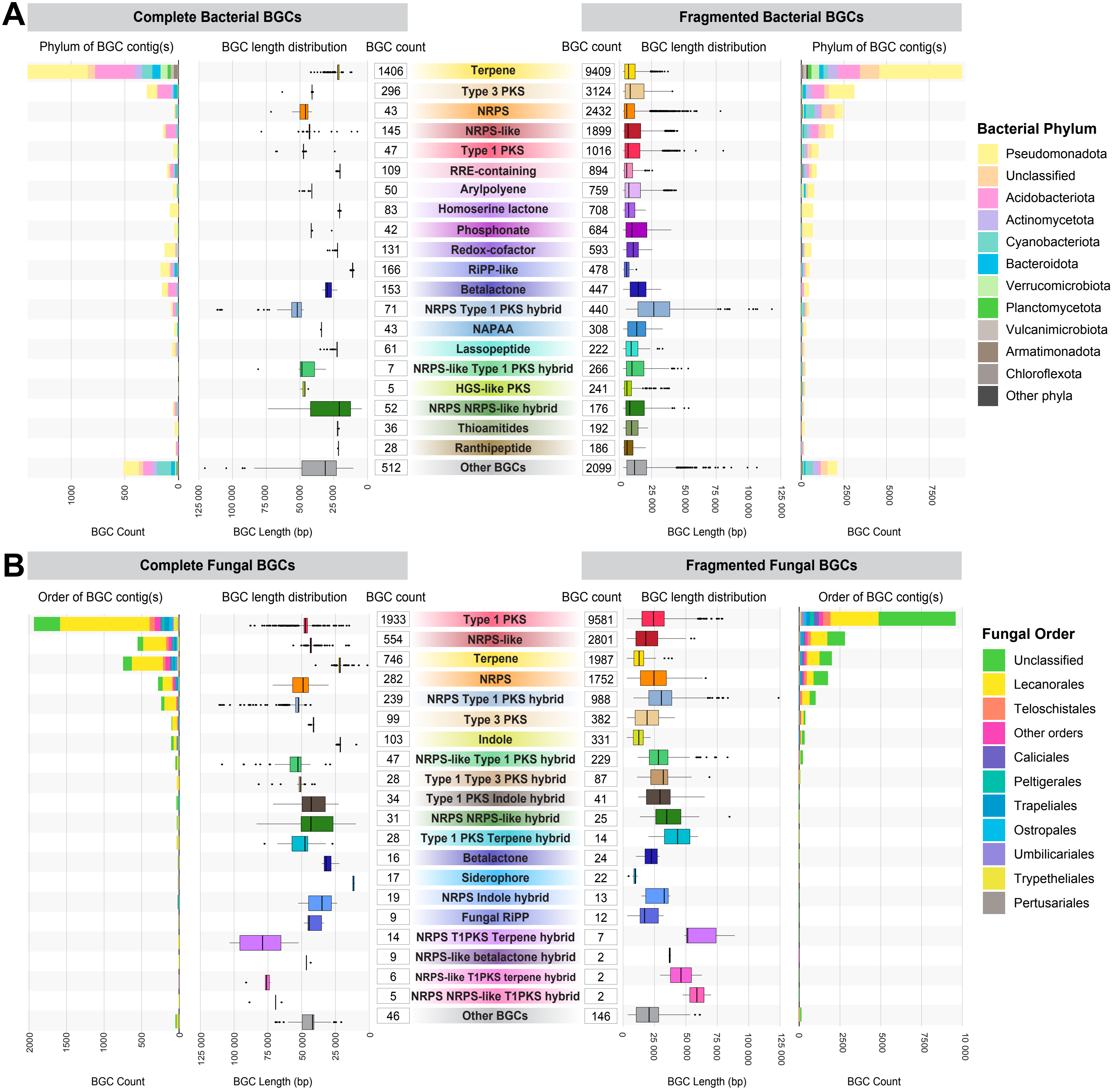

A
Complete Bacterial BGCs
Fragmented Bacterial BGCs
Phylum of BGC contig(s)
BGC length distribution
BGC count
BGC count
BGC length distribution
Phylum of BGC contig(s)
1406 Terpene 9409
296 Type 3 PKS 3124
43 NRPS 2432
145 NRPS-like 1899
47 Type 1 PKS 1016
109 RRE-containing 894
50 Arylpolyene 759
83 Homoserine lactone 708
42 Phosphonate 684
131 Redox-cofactor 593
166 RiPP-like 478
153 Betalactone 447
71 NRPS Type 1 PKS hybrid 440
43 NAPAA 308
61 Lassopeptide 222
7 NRPS-like Type 1 PKS hybrid 266
5 HGS-like PKS 241
52 NRPS NRPS-like hybrid 176
36 Thioamitides 192
28 Ranthipeptide 186
512 Other BGCs 2099
1000
500
0
125 000
100 000
75 000
50 000
25 000
0
0
25 000
50 000
75 000
100 000
125 000
0
2500
5000
7500
BGC Count
BGC Length (bp)
BGC Length (bp)
BGC Count
Bacterial Phylum
Pseudomonadota
Unclassified
Acidobacteriota
Actinomycetota
Cyanobacteriota
Bacteroidota
Verrucomicrobiota
Planctomycetota
Vulcanimicrobiota
Armatimonadota
Chloroflexota
Other phyla
B
Complete Fungal BGCs
Fragmented Fungal BGCs
Order of BGC contig(s)
BGC length distribution
BGC count
BGC count
BGC length distribution
Order of BGC contig(s)
1933 Type 1 PKS 9581
554 NRPS-like 2801
746 Terpene 1987
282 NRPS 1752
239 NRPS Type 1 PKS hybrid 988
99 Type 3 PKS 382
103 Indole 331
47 NRPS-like Type 1 PKS hybrid 229
28 Type 1 Type 3 PKS hybrid 87
34 Type 1 PKS Indole hybrid 41
31 NRPS NRPS-like hybrid 25
28 Type 1 PKS Terpene hybrid 14
16 Betalactone 24
17 Siderophore 22
19 NRPS Indole hybrid 13
9 Fungal RiPP 12
14 NRPS T1PKS Terpene hybrid 7
9 NRPS-like betalactone hybrid 2
6 NRPS-like T1PKS terpene hybrid 2
5 NRPS NRPS-like T1PKS hybrid 2
46 Other BGCs 146
2000
1500
1000
500
0
125 000
100 000
75 000
50 000
25 000
0
0
25 000
50 000
75 000
100 000
125 000
0
2500
5000
7500
10 000
BGC Count
BGC Length (bp)
BGC Length (bp)
BGC Count
Fungal Order
Unclassified
Lecanorales
Teloschistales
Other orders
Caliciales
Peltigerales
Trapeliales
Ostropales
Umbilicariales
Trypetheliales
Pertusariales
