# Supplementary material for: A Biosynthetic and Taxonomic Atlas of the Global Lichen Holobiont
Source: Environ Microbiol. 2025 Jun 4;27(6):e70112. doi: 10.1111/1462-2920.70112 (PMC12136951; doi:10.1111/1462-2920.70112)
Supplement: Supplementary file 3 — FIGURE S3. Bacterial MAGS recovered from 794 lichen holobiont metagenomes. (A) The quality distribution of MAGs across all samples. (B) The taxonomic distribution of high and medium quality MAGs at the phylum level from all samples. [file EMI-27-e70112-s006.pdf]

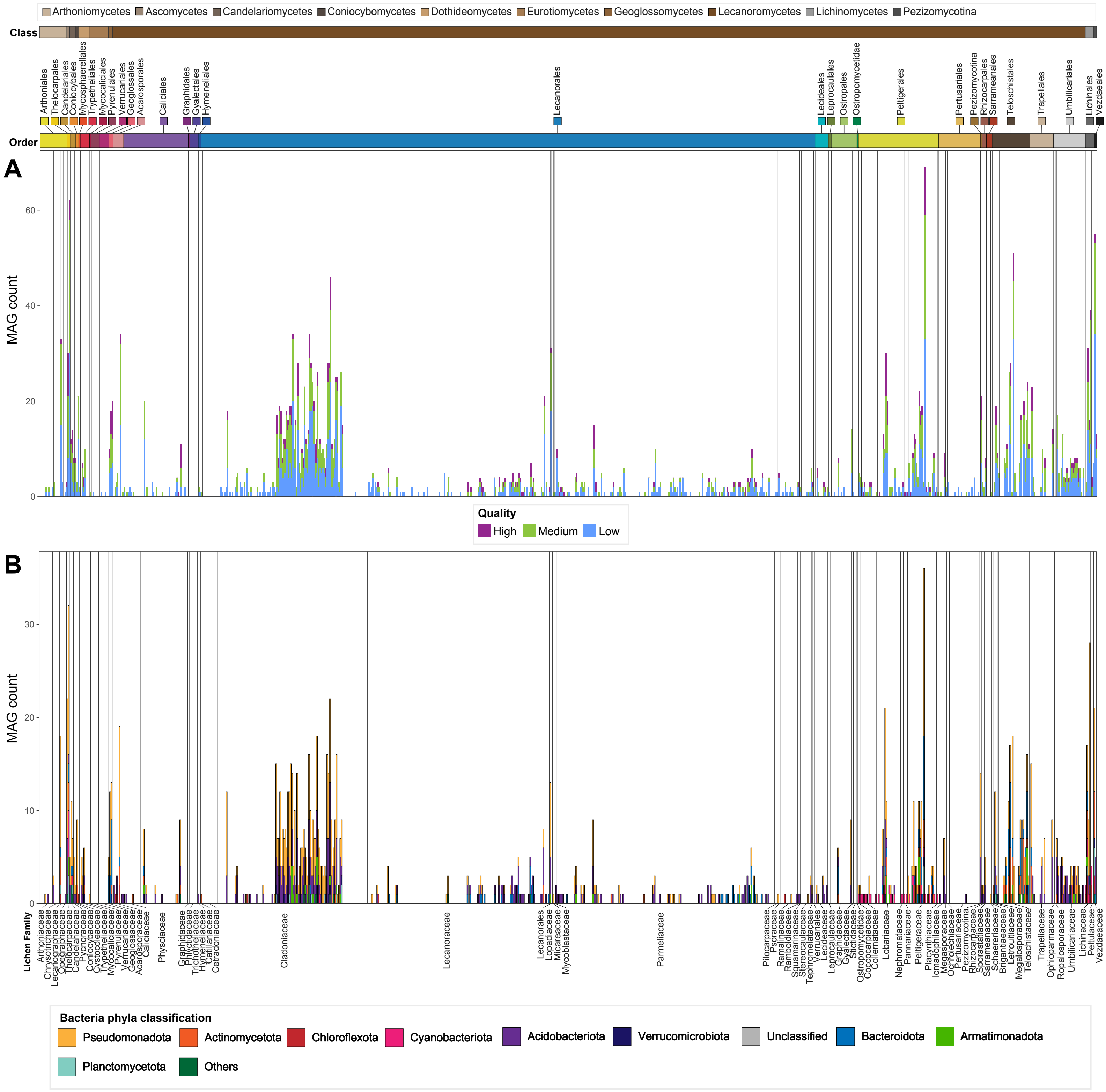
Arthoniomycetes
Ascomycetes
Candelariomycetes
Coniocybomycetes
Dothideomycetes
Eurotiomycetes
Geoglossomycetes
Lecanoromycetes
Lichinomycetes
Pezizomycotina
Class
Arthoniales
Thelocarpales
Candelariales
Coniocybales
Mycosphaerellales
Trypetheliales
Mycocaliciales
Pyrenulales
Verrucariales
Geoglossales
Acarosporales
Caliciales
Graphidales
Gyalectales
Hymeneliales
Lecanorales
Lecideales
Leprocaulales
Ostropales
Ostropomycetidae
Peltigerales
Pertusariales
Pezizomycotina
Rhizocarpales
Sarrameanales
Teloschistales
Trapeliales
Umbilicariales
Lichinales
Vezdaeales
Order
A
MAG count
0
20
40
60
Quality
High
Medium
Low
B
MAG count
0
10
20
30
Lichen Family
Arthoniaceae
Chrysotrichaceae
Lecanographaceae
Opegraphaceae
Thelocarpaceae
Candelariaceae
Pycnoraceae
Coniocybaceae
Cystocoleaceae
Trypetheliaceae
Mycocaliciaceae
Pyrenulaceae
Verrucariaceae
Geoglossaceae
Acarosporaceae
Caliciaceae
Physciaceae
Graphidaceae
Phlyctidaceae
Trichotheliaceae
Hymeneliaceae
Catillariaceae
Cetradoniaceae
Cladoniaceae
Lecanoraceae
Lecanorales
Lopadiaceae
Micareaceae
Mycoblastaceae
Parmeliaceae
Pilocarpaceae
Psoraceae
Ramalinaceae
Ramboldiaceae
Squamarinaceae
Stereocaulaceae
Tephromelataceae
Verrucariales
Lecideaceae
Leprocaulaceae
Graphidaceae
Gyalectaceae
Stictidaceae
Ostropomycetidae
Coccocarpiaceae
Collemataceae
Lobariaceae
Nephromataceae
Pannariaceae
Peltigeraceae
Placynthiaceae
Icmadophilaceae
Megasporaceae
Ochrolechiaceae
Pertusariaceae
Pezizomycotina
Rhizocarpaceae
Sporastatiaceae
Sarrameanaceae
Schaereriaceae
Brigantiaeaceae
Letrouitiaceae
Megalosporaceae
Teloschistaceae
Trapeliaceae
Ophioparmaceae
Ropalosporaceae
Umbilicariaceae
Lichinaceae
Peltulaceae
Vezdaeaceae
Bacteria phyla classification
Pseudomonadota
Actinomycetota
Chloroflexota
Cyanobacteriota
Acidobacteriota
Verrucomicrobiota
Unclassified
Bacteroidota
Armatimonadota
Planctomycetota
Others
